# Supplementary material for: Inhibition of adenovirus replication by CRISPR-Cas9-mediated targeting of the viral E1A gene
Source: Mol Ther Nucleic Acids. 2023 Mar 3;32:48–60. doi: 10.1016/j.omtn.2023.02.033 (PMC10025986; doi:10.1016/j.omtn.2023.02.033)
Supplement: Document S1. Figures S1–S7 and Tables S1 and S2 [file mmc1.pdf]

OMTN, Volume *32*

Supplemental information

Inhibition of adenovirus replication by CRISPR-Cas9-mediated targeting of the viral E1A gene

Zrinka Didara, Florian Reithofer, Karina Zöttl, Alexander Jürets, Izabella Kiss, Angela Witte, and Reinhard Klein

Table S1: Primer List

Primer names	5´-3´
pENTR4 FW	GGAAAGAACCGGCGCGCCAAGCTTGAATTCGCGGCCGCACTC
pENTR4 RV	AAGCAGATTCGACTGAATTGGTTCCCATGGTG
CMV Tet02_FW	CAATTCAGTCGAATCTGCTTAGGGTTAG
CMV Tet02_FW	ATAGTGAGTCGTTTAAACGCTAGAGTCC
Cas9 HF1 FW	GCGTTTAAACGACTCACTATAGGGAGAGCC
Cas9 HF1 RV	TTGGCGCGCCGGTTCTTTCCGCCTCAGAAG
RV01 sequencing FW	GTTCCAGTACGGCTCCAAG
RV01 sequencing RV	CTGGCGGCCGCTTTACTTG
T7E1 E1A Set1 FW	CAGCGAGTAGAGTTTTCTCC
T7E1 E1A Set1 RW	GTAGACAAACATGCCACAGG
T7E1 E1A Set2 FW	CGGTGAGTTCCTCAAGAG
T7E1 E1A Set2 RW	CCAAACCCACCACTCTATC
T7E1 E1A Set3 FW	GTCAGCTGACGTGTAGTG
T7E1 E1A Set3 RW	CCGTACTACTATTGCATTCTCTAG
E3 qPCR FW	TGCTGCACTGCTATGCTAAT
E3 qPCR RV	TCCTCAATAAAGCTGCGTCTG
E3 qPCR probe	TGCTCGCTTTGGTCTGTACCCTAC
E1A/E1B loop seq FW	TACCCGGTGAGTTCCTCAAG
E1A/E1B loop seq RV	GCACCCATCCCAGCTTAACC
Cas9 qPCR FW	ACGGGATAAGAGACAAGCAAAG
Cas9 qPCR RV	GGTTAAAGAGTCATCATGGATCAG
Cas9HFqPCR_probe	TAAAGAGCGACGGCTTCGCCAATA
OTUD5 F1	ATGAGAGAGAGTGCAGGGGT
OTUD5 R1	TCACAGGCCTAGCAGATCCT
IFFO1 F1	GCTCCCACCTCCCTTCAAAA
IFFO1 R1	GTGGGTGTCTGCTACTTCCC
GPR85 F1	GTGTGCTCAGTCCAAGAGGG
GPR85 R1	TGAGGAGTCAAGAGCAACGG
UNC80 F2	GCCATTTTCAGAGGAGGACA
UNC80 R2	ACGTATGCAAGAGGACACACC
RNF111 F1	CGTGGCACTATCACATTATTTACAG
RNF111 R1	GTCTTTCCACAGGGCAAGCA
FOXJ2 F2	GGAGAGGCCACATTACCAAG
FOXJ2 R2	TTGTCCTCATGCCCTACCAT
CTD-3060P21.1/RAP1GAP2 F1	CTGCCTGCTGCTGTCTTAGT
CTD-3060P21.1/RAP1GAP2 R1	TCCGGAATCTGCCCTCAGTA

TOB1-AS1 F1	GTCTGGAAAGGGACTGTGGG
TOB1-AS1 R1	TAAACGGATCCGAGTCGCAG
ABCF3 F1	CCTGAGGAGGAGTACCGTCA
ABCF3 R1	CTGAAAGACAGGAGGGCAGG
TMEM110 F1	CACCACCACCTGGATCTCAC
TMEM110 R1	TGAGAGCAGATCAGAGGGCT
PRKACB F1	CCACCATGGTGTTCTGGAGG
PRKACB R1	TGTAGCATCAAAAGAAGGCCA
RP5-964N17.1 F1	TGCTGTCTAAGTTCTGCGCC
RP5-964N17.1 R1	GCCCAGTCAGCTACAGAGTG

Table S2: Surrogate Reporter Vector Target Sequences

Vector name	Target sequence inserted into RV01	5´-3´
RVO1-1,3,4	E1A gRNA 1,3,4	GAATTCAACCTTGTACCGGAGGTGATCGATCTTACCTGCCACGAGGCTGGCTGGATCC
RVO1-2,9	E1A gRNA 2,9	GAATTCCCCCCGAAGATCCCAACGAGGAGGCGTGTAATGTTGGCGGTGCAGGAAGGGAGGATCC
RVO1-5,6,8	E1A gRNA 5,6,8	GAATTCGCCAGTCTTTTGGACCAGCTGATCGTCCACCTCCTAGCCATTTTGAACCACCTACGGATCC
RVO1-7,10	E1A gRNA 7,10	GAATTCGCCCGGTTCTCCGGAGCCGCCTCACCTTTCCCGGCAGCCCGAGCAGCCGGGATCC

Figure S1

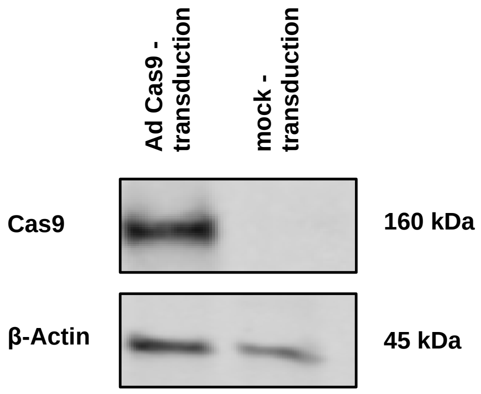

Figure S1. Expression of spCas9-HF1.

The functionality of the spHF-Cas9 expression cassettes harboring the doxycycline-regulatable CMV promoter which is present in all adenoviral CRISPR/Cas9 vectors was proven in the absence of doxycycline in HeLa cells lacking the tetracycline repressor. Equal amounts of protein from HeLa cells at 48 h post-transduction were subjected to Western blot analysis for the detection of spHF-Cas9 and for comparison of □-actin. The expression is exemplarily shown for the vector expressing only spCas9-HF1.

Figure S2

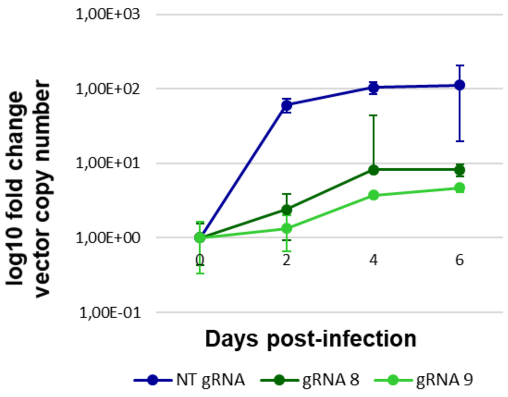

Figure S2. Replication of the adenoviral vectors in HAdV-5-infected cells.

HeLa cells were transduced with the adenoviral vectors containing Cas9 in combination with the targeting gRNAs 8 or 9 or with a non-targeting (NT) gRNA at an MOI of 30. 24 h after transduction the cells were infected with HAdV-5 at an MOI of 0.1. Vector copy numbers at time points 0, 2, 4, and 6 days post-infection were determined by qPCR with primers/probe specific for the Cas9-encoding part of the vectors. Data represent the means (n = 3) ± SD of triplicate infections of a representative experiment.

Figure S3

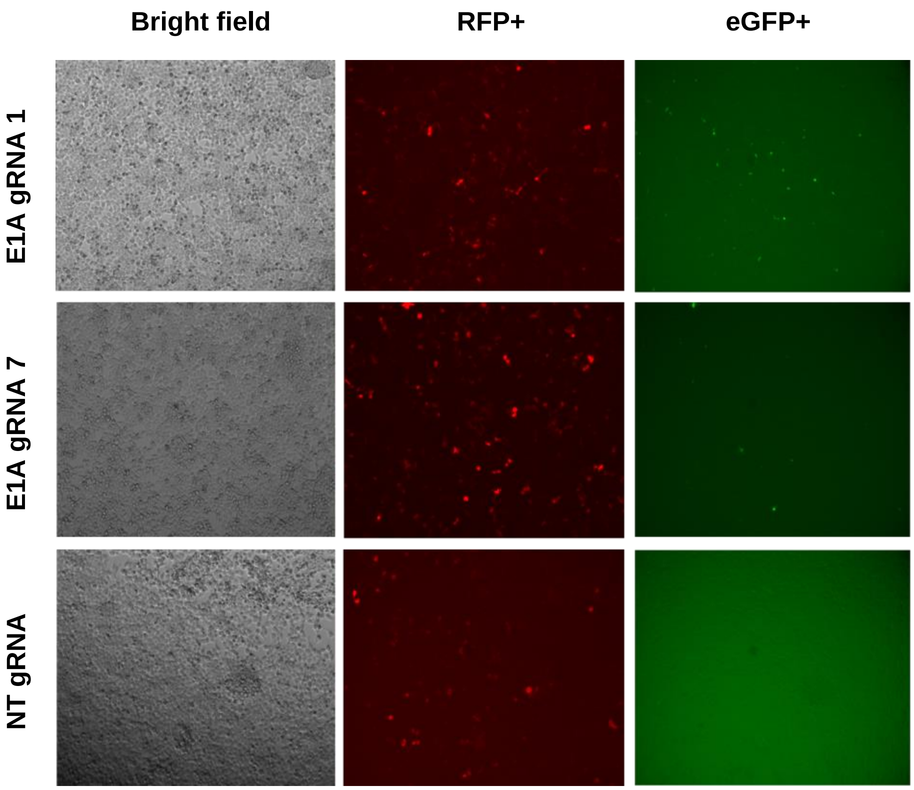

Figure S3. Detection of gene editing by E1A-targeting gRNAs 1 and 7 in surrogate reporter assays.

HEK293 cells were transfected with the surrogate reporter vector and transduced with the recombinant adenoviral vectors expressing either a targeting or a non-targeting (NT) gRNA. Fluorescence was monitored 48h post-transduction with a Leica DMi8 System. Bright field, red and green fluorescence images at a magnification of 10x are shown. Microscopy settings were: HC PL FLUOTAR CS 10x/0.40 DRY; Camera Leica DFC 360FX: active resolution 1392 x 1040, pixel bitdepth 12/8 bit, pixel size 6.45 µm x 6.45 µm; live image with 1392 x 1040 at 20 images/second.

Figure S4

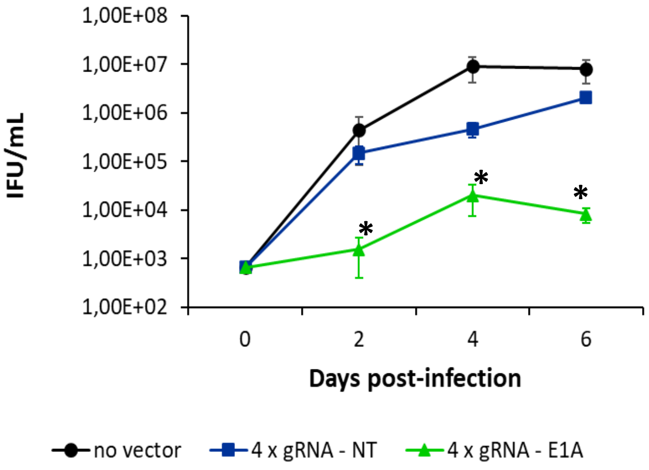

Figure S4. Inhibition of HAdV-5 replication in A549 cells.

A549 cells were transduced with the adenoviral vector containing Cas9 in combination with gRNAs 1, 7, 8, and 9 expressed from individual promoters or with a control vector carrying four non-targeting (NT) gRNAs at an MOI of 100 followed by infection of the cells with HAdV-5 at an MOI of 0.01 24 h later. Numbers of infectious virus particles were determined at the indicated time points and were expressed as IFU/mL (infectious units per mL). Data represent the means (n = 3) ± SD of three infections. *$p < 0.05$; *** $p < 0.001$.

Figure S5

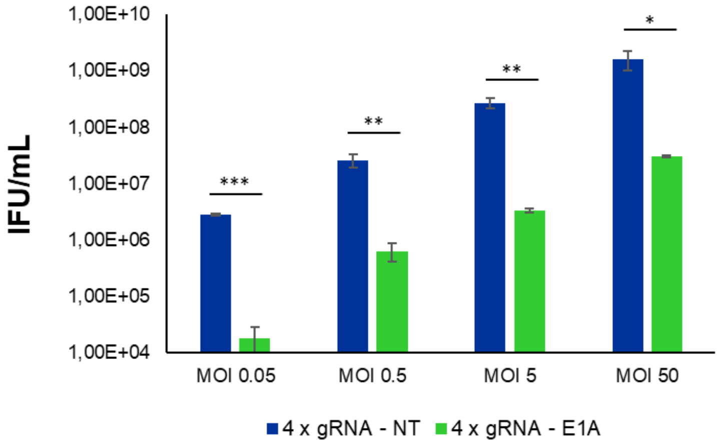

Figure S5. Inhibition of HAdV-5 replication at increased MOIs.

A549 cells were transduced with the adenoviral vectors containing Cas9 in combination with the E1A-targeting gRNAs 1, 7, 8, and 9 expressed from individual promoters or with a corresponding control vector carrying four non-targeting (NT) gRNAs instead of the targeting gRNAs at a constant MOI of 50. 24 h after transduction the cells were infected with HAdV-5 at increasing MOIs ranging from 0.05 to 50. 48 h after infection numbers of infectious virus particles were determined and were expressed as IFU/mL (infectious units per mL). Data represent the means (n = 3) ± SD of triplicate infections of a representative experiment. *$p < 0.05$; ** $p < 0.01$; *** $p < 0.001$.

Figure S6

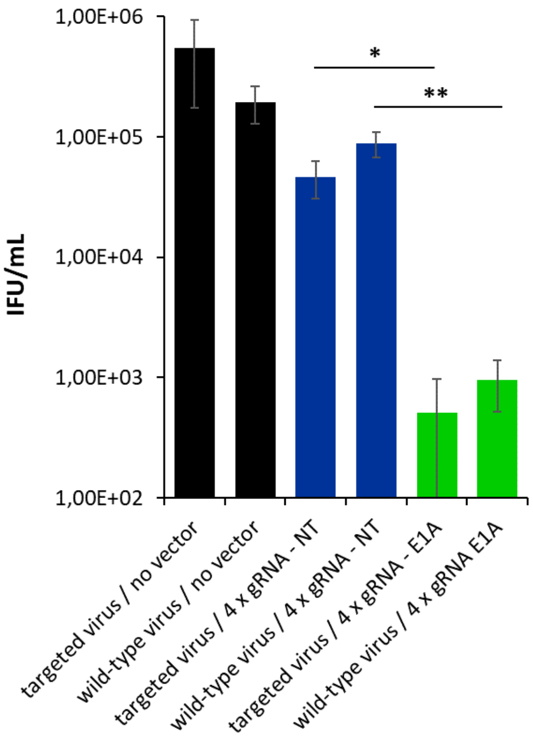

Figure S6. CRISPR/Cas9-mediated inhibition of replication of virus recovered from the first round of targeting by CRISPR/Cas9

HeLa cells were transduced with the adenoviral vector containing Cas9 in combination with gRNAs 1, 7, 8, and 9 expressed from individual promoters (green), with a control vector carrying four non-targeting (NT) gRNAs (blue), or were mock-transduced (black) at an MOI of 100. 24 h later cells were infected with virus recovered after the first round of targeting by CRISPR/Cas9 (day 6 time point; virus pooled from three independent experiments) or with non-targeted wild-type virus at an MOI of 0.01. Virus was allowed to replicate for two days. At day 2 post-infection numbers of infectious virus particles were determined and were expressed as IFU/mL (infectious units per mL). Data represent the means (n = 3) ± SD of three infections. *$p < 0.05$, **$p < 0.01$.

Figure S7

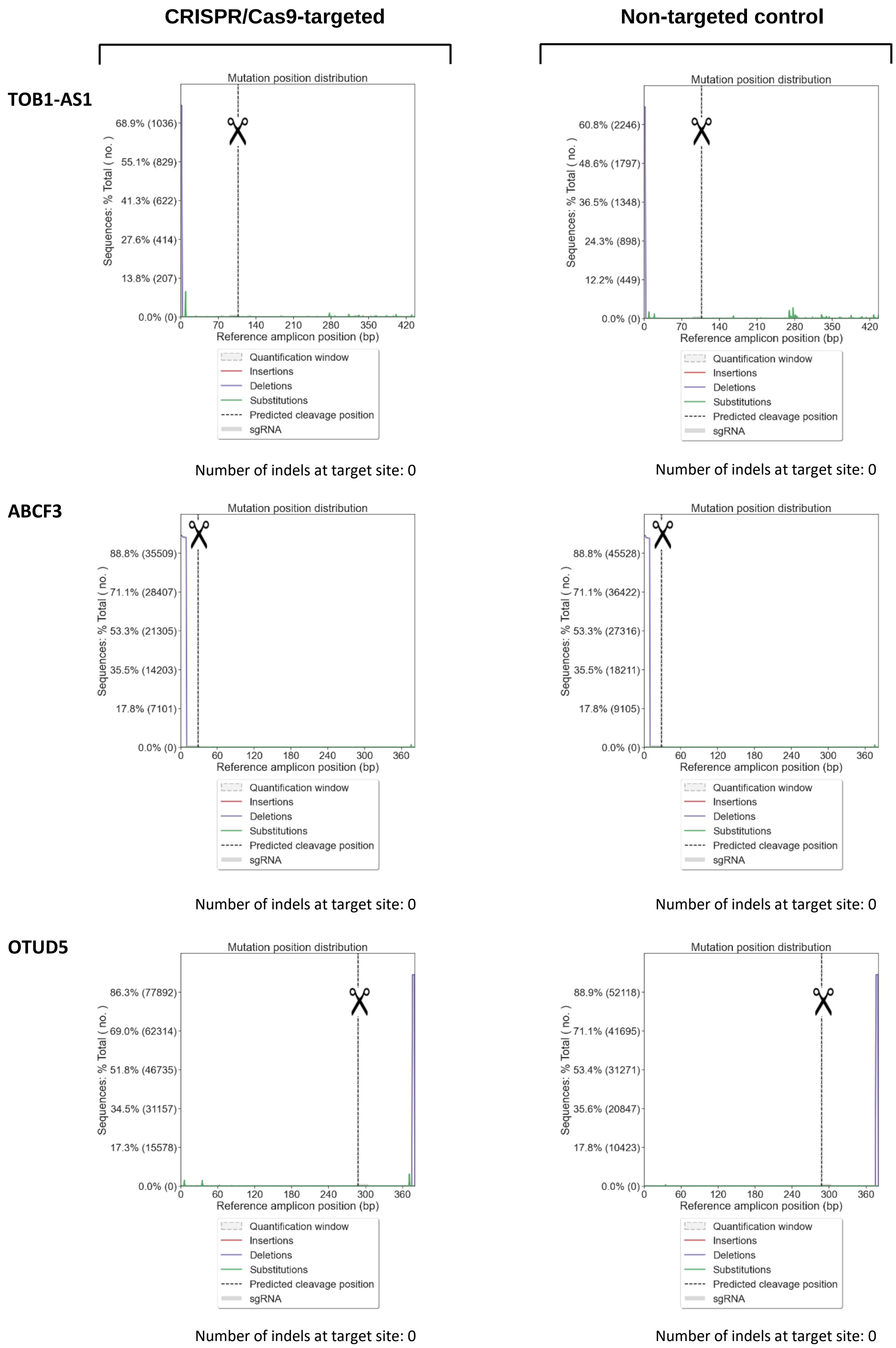
CRISPR/Cas9-targeted
Non-targeted control
TOB1-AS1
Mutation position distribution
68.9% (1036)
55.1% (829)
41.3% (622)
27.6% (414)
13.8% (207)
0.0% (0)
Sequences: % Total (no.)
0
70
140
210
280
350
420
Reference amplicon position (bp)
Quantification window
Insertions
Deletions
Substitutions
Predicted cleavage position
sgRNA
Number of indels at target site: 0
Mutation position distribution
60.8% (2246)
48.6% (1797)
36.5% (1348)
24.3% (898)
12.2% (449)
0.0% (0)
Sequences: % Total (no.)
Reference amplicon position (bp)
Number of indels at target site: 0
ABCF3
Mutation position distribution
88.8% (35509)
71.1% (28407)
53.3% (21305)
35.5% (14203)
17.8% (7101)
0.0% (0)
Sequences: % Total (no.)
0
60
120
180
240
300
360
Reference amplicon position (bp)
Number of indels at target site: 0
Mutation position distribution
88.8% (45528)
71.1% (36422)
53.3% (27316)
35.5% (18211)
17.8% (9105)
0.0% (0)
Sequences: % Total (no.)
Reference amplicon position (bp)
Number of indels at target site: 0
OTUD5
Mutation position distribution
86.3% (77892)
69.0% (62314)
51.8% (46735)
34.5% (31157)
17.3% (15578)
0.0% (0)
Sequences: % Total (no.)
Reference amplicon position (bp)
Number of indels at target site: 0
Mutation position distribution
88.9% (52118)
71.1% (41695)
53.4% (31271)
35.6% (20847)
17.8% (10423)
0.0% (0)
Sequences: % Total (no.)
Reference amplicon position (bp)
Number of indels at target site: 0

Figure S7 - continued

CRISPR/Cas9-targeted | **Non-targeted control**

IFFO1

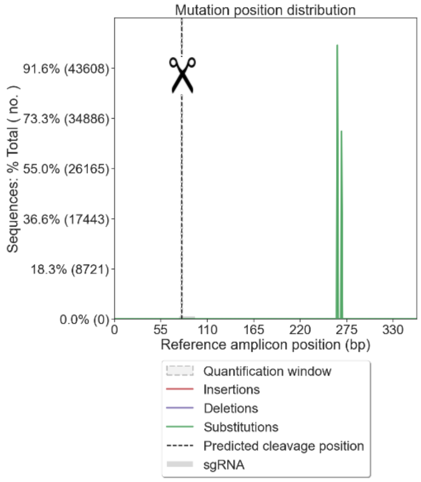

Number of indels at target site: 0

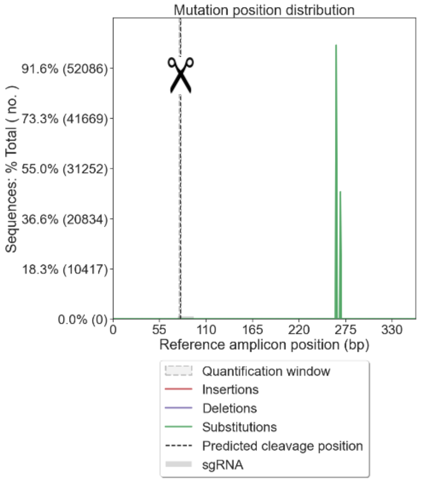

Number of indels at target site: 0

RP5-964N17.1

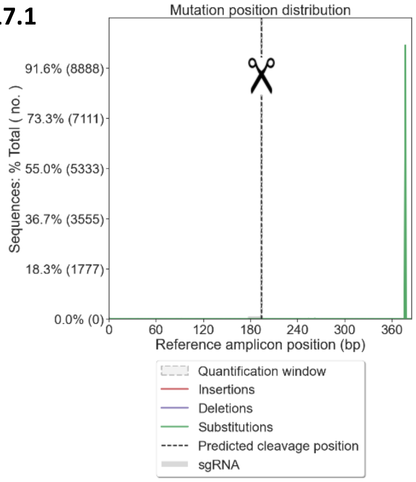

Number of indels at target site: 0

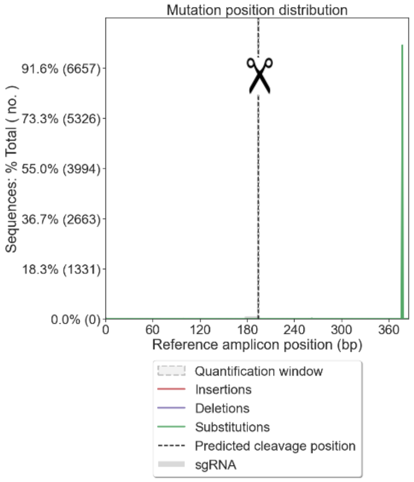

Number of indels at target site: 0

GPR85

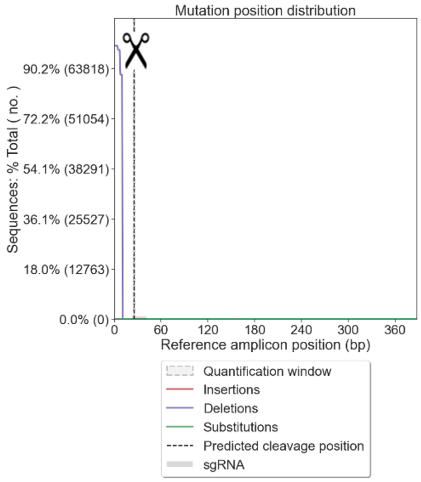

Number of indels at target site: 0

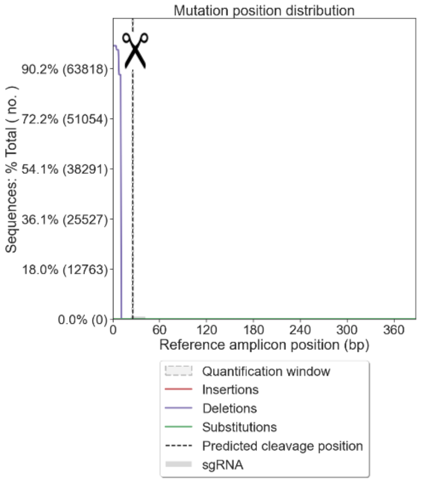

Number of indels at target site: 0

Figure S7 - continued

CRISPR/Cas9-targeted | **Non-targeted control**

PRKACB

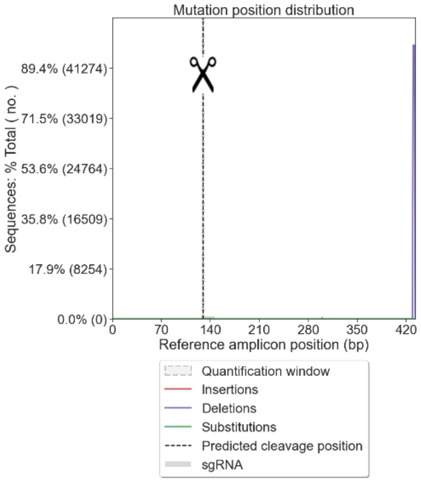

Number of indels at target site: 0

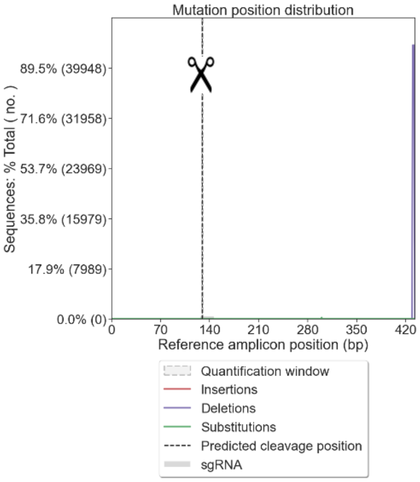

Number of indels at target site: 0

TMEM110

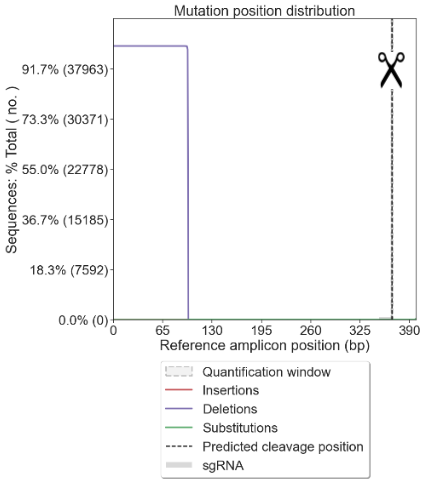

Number of indels at target site: 0

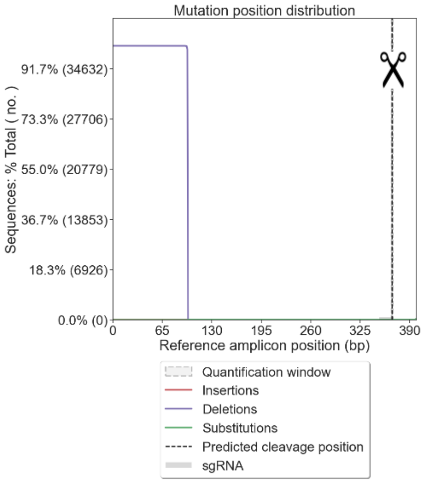

Number of indels at target site: 0

UNC80

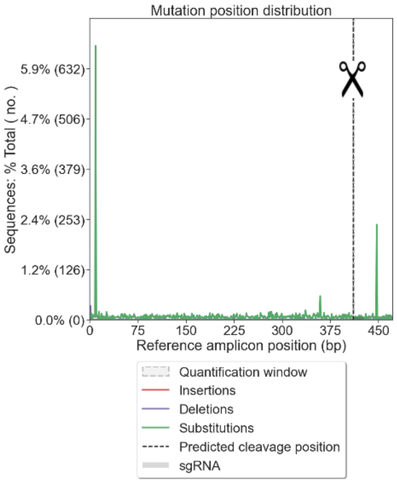

Number of indels at target site: 0

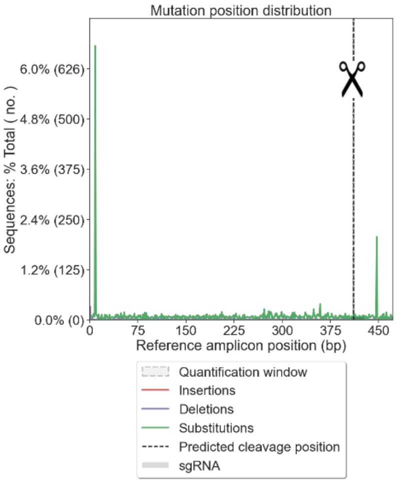

Number of indels at target site: 0

Figure S7 - continued

CRISPR/Cas9-targeted | **Non-targeted control**

RNF111

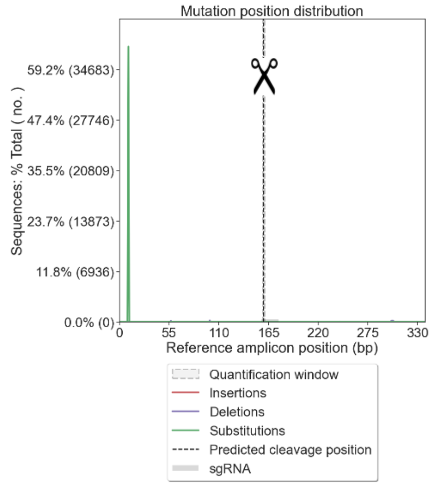

Number of indels at target site: 0

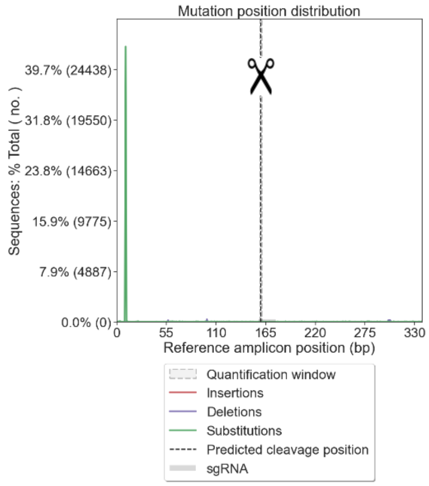

Number of indels at target site: 0

FOXJ2

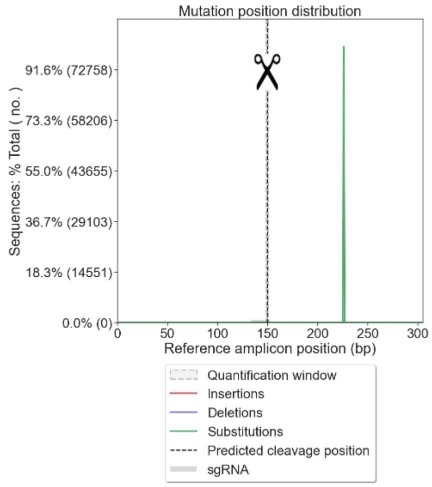

Number of indels at target site: 0

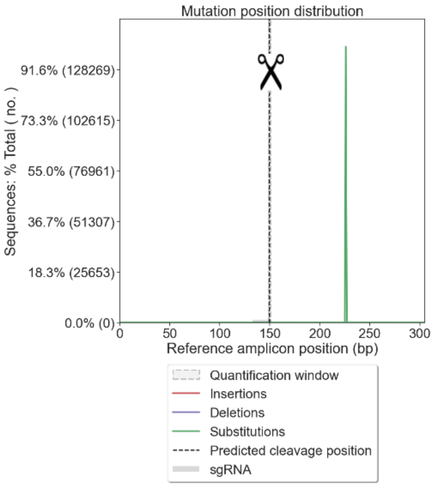

Number of indels at target site: 0

CTD-3060P21.1/

RAP1GAP2

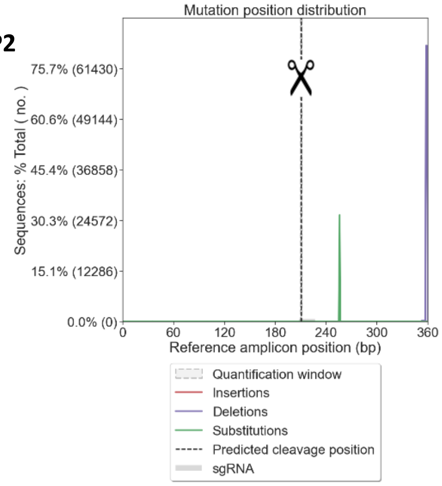

Number of indels at target site: 0

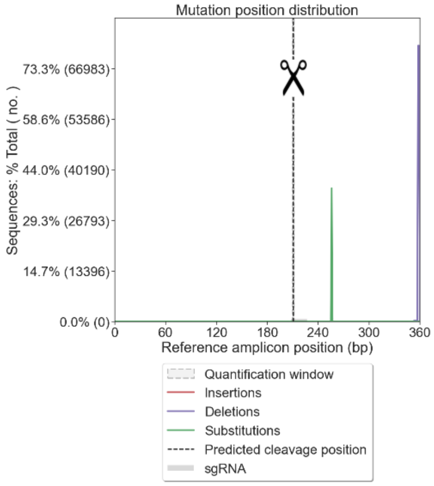

Number of indels at target site: 0

Figure S7. CRISPR/Cas9-mediated cleavage at potential off-target sites.

HeLa cells were transduced with the adenoviral vectors harboring Cas9 in combination with the four gRNAs 1, 7, 8 and 9 expressed from separate promoters at an MOI of 100 or were mock-transduced. 24 h after transduction the cells were infected with HAdV-5 at an MOI of 0.01. Two days post-infection DNA was isolated from the cells, the DNA regions comprising the potential off-target sites were amplified by PCR, the amplicons were subjected to next generation sequencing, and the sequencing reads were mapped to the respective reference amplicon sequences. Percentages of modifications around the indicated potential off-target sites in cells targeted with CRISPR/Cas9 (left) and in non-targeted control cells (right) representing background noise as a result of randomly occurring PCR amplification/sequencing were calculated with CRISPResso2. Insertions, deletions, and substitutions are indicated. Deletions (blue) appearing close to the borders of the graphs do not represent CRISPR/Cas9-mediated modifications but indicate the ends of the amplicons that were not covered by sequencing. Substitutions (green) occurring at more or less the same frequencies at the same positions in targeted and non-targeted cells represent mutations that have accumulated in the HeLa cell population that was used for the experiments. The potential cleavage sites at which modifications would appear in case of off-targeting are indicated with dashed lines and pairs of scissors.
