# Supplementary material for: Inhibition of adenovirus replication by CRISPR-Cas9-mediated targeting of the viral E1A gene
Source: Mol Ther Nucleic Acids. 2023 Mar 3;32:48–60. doi: 10.1016/j.omtn.2023.02.033 (PMC10025986; doi:10.1016/j.omtn.2023.02.033)
Supplement: Document S2. Article plus supplemental information [file mmc2.pdf]

Molecular Therapy
Nucleic Acids
Original Article

Inhibition of adenovirus replication by CRISPR-Cas9-mediated targeting of the viral E1A gene

Zrinka Didara,[1,3] Florian Reithofer,[1,3] Karina Zöttl,[1] Alexander Jürets,[1] Izabella Kiss,[1] Angela Witte,[2] and Reinhard Klein[1]

[1]Department of Life Sciences, University of Applied Sciences Krems, Piaristengasse 1, 3500 Krems, Austria; [2]Department of Microbiology, Immunobiology, and Genetics, Max Perutz Labs, University of Vienna, Dr. Bohr-Gasse 9, 1030 Vienna, Austria

DNA-targeting CRISPR-Cas systems are able to cleave dsDNA in mammalian cells. Accordingly, they have been employed to target the genomes of dsDNA viruses, mostly when present in cells in a non-replicative state with low copy numbers. However, the sheer amount of viral DNA produced within a very short time by certain lytically replicating viruses potentially brings the capacities of CRISPR-Cas systems to their limits. The accessibility of viral DNA replication sites, short time of accessibility of the DNA before encapsidation, or its complexation with shielding proteins are further potential hurdles. Adenoviruses are fast-replicating dsDNA viruses for which no approved antiviral therapy currently exists. We evaluated the potency of CRISPR-Cas9 in inhibiting the replication of human adenovirus 5 *in vitro* by targeting its master regulator *E1A* with a set of guide RNAs and observed a decrease in infectious virus particles by up to three orders of magnitude. Target DNA cleavage also negatively impacted the amount of viral DNA accumulated during the infection cycle. This outcome was mainly caused by specific deletions, inversions, and duplications occurring between target sites, which abolished most E1A functions in most cases. Additionally, we compared two strategies for multiplex gRNA expression and obtained comparable results.

INTRODUCTION

After their discovery, DNA-recognizing CRISPR-Cas systems[1] were rapidly developed into tools for editing cellular DNA.[2–4] In the engineered versions, a single guide RNA (gRNA) pilots Cas9 to the target sequence residing adjacent to a so-called "protospacer adjacent motif" (PAM; 5′-NGG-3′), followed by cleavage of the dsDNA and repair of the break via non-homologous end joining (NHEJ). As a result, small insertions or deletions (indels) occur at the cleavage site, which can result in frameshifts that render the affected protein inactive. In addition, the homology-directed repair (HDR) pathway is activated and can be exploited to insert DNA into target sites. CRISPR-Cas systems have also been evaluated for inactivation of the (pro)viral DNA of viruses such as human immunodeficiency virus (HIV), simian immunodeficiency virus (SIV), hepatitis B virus (HBV), herpesviruses, and human papillomavirus (HPV).[5,6] However, in most of these cases, the viral DNA was targeted while present in a non-replicative state, either incorporated into the host chromosome or present in a stable extrachromosomal form with low copy numbers. In these studies, the main goal was to eradicate viral genomes from infected cells because they resist elimination by conventional drugs.

Targeting of actively replicating DNA viruses may potentially be complicated by several factors: (1) the viral DNA must be in a state that allows recognition by CRISPR-Cas9 (i.e., it must not be complexed with proteins that prevent the interaction), (2) spatial separation of the viral replication sites and the CRISPR-Cas9 effectors might make contact between them impossible, (3) the time span during which the CRISPR-Cas9 components have access to the viral DNA before it is encapsidated again might be too short, and (4) the inherent CRISPR-Cas9 potency might not be sufficient to allow these systems to cope with the sheer amount of DNA produced by certain viruses during lytic infection. To date, only a few DNA viruses have been investigated for targeting by CRISPR-Cas9 during lytic infection. These include vaccinia virus,[7] African swine fever virus,[8] polyomavirus JC,[9,10] and a few examples of *de novo* infection of cells with viruses that are primarily investigated for being targeted during persistent infections, such as herpesviruses and HBV, for example.[11–15]

Adenoviruses[16,17] contain dsDNA genomes, which renders them suitable for targeting by CRISPR-Cas9. Human adenoviruses are associated with a variety of clinical symptoms mostly affecting the respiratory and intestinal tracts but also the eyes. Infections are mostly self-limiting but can become serious and even life-threatening in immunocompromised patients.[18–21] Currently, no approved antiviral therapies for adenoviruses exist; hence, the treatment relies on repurposing drugs for treatment of other viral diseases,[22] such as

Received 17 August 2022; accepted 28 February 2023;
https://doi.org/10.1016/j.omtn.2023.02.033.

[3]These authors contributed equally

Correspondence: Reinhard Klein, Department of Life Sciences, University of Applied Sciences Krems, Piaristengasse 1, 3500 Krems, Austria.
E-mail: reinhard.klein@fh-krems.ac.at

 © 2023 The Author(s).
This is an open access article under the CC BY license (http://creativecommons.org/licenses/by/4.0/).

cidofovir (CDV) and derivatives, which, however, show limited efficacy, cause toxicity, or are still under investigation.[23–26] Thus, alternative treatment options are needed, and it is theoretically conceivable to develop CRISPR-Cas9 into a therapeutic agent to treat localized infections, such as infections of the eye, or even disseminated infections. In such therapeutic scenarios, delivery may be based on viral or non-viral DNA or RNA.

In permissive cells, adenoviruses multiply productively and lyse their hosts. Fast-replicating human adenoviruses, such as human adenovirus 5 (HAdV-5), reach burst sizes of 1e+04 infectious units per cell or more. The central viral regulator of the infection cycle is the early region 1A (*E1A*) gene,[27–32] the first gene to be expressed after infection, which is needed to transactivate the expression of other viral genes.[33] In addition, E1A interacts with a large number of cellular targets,[30] manipulating their function with broad consequences for the cellular transcriptome, proteome, and interactome to create an environment that is beneficial for the virus.[30,31] Most E1A functions reside in four conserved regions (CR1–CR4).[34] Differential splicing of *E1A* RNA gives rise to several isoforms, of which the two largest ones (E1A 289R and E1A 243R) execute most of the functions. Smaller isoforms accumulate during the late phase of infection, but their function is less well understood.[35,36] Because of its central role in the infection cycle, *E1A* constitutes a conceivable target for CRISPR-Cas9-based inhibition of adenovirus replication.

In this study, we provide a proof of principle showing that adenoviral DNA is amenable to recognition by CRISPR-Cas9 and that the time frame during which the viral DNA is accessible is large enough to allow efficient inhibition of virus replication by up to three orders of magnitude when the viral *E1A* gene is targeted. Our data suggest that CRISPR-Cas9 has an intrinsic ability to cope with the sheer numbers of viral DNA molecules generated during lytic adenovirus infection, provided that efficient delivery/production of CRISPR-Cas9 effectors is ensured.

RESULTS

Selection of adenovirus-targeting gRNAs and delivery of CRISPR-Cas9 effectors

To evaluate the potential of CRISPR-Cas9 to inhibit multiplication of lytically replicating adenoviruses, we chose HAdV-5 as a model system and the E1A gene as the target. We selected 10 gRNAs predicted to bind to the left half of *E1A* (Figure 1), comprising the functionally important CR1 and CR2, both of which are part of the dominating E1A isoforms E1A 289R and E1A 243R. As a Cas9 effector, we used a high-fidelity version of Cas9, spCas9-HF1, which has greatly reduced off-target cleavage activity.[37] For delivery, we employed replication-deficient, E1- and E3-deleted, HAdV-5-based vectors because these vectors ensure efficient delivery into target cells *in vitro* and are amplified in cells infected with HAdV-5, increasing the copy number of CRISPR-Cas9 effector-encoding sequences in these cells. We generated vectors containing the expression cassettes for Cas9 alone and Cas9 together with individual targeting gRNAs or a non-targeting control gRNA (Figure 2). These vectors need to be amplified in HEK293 cells expressing adenoviral *E1A* for complementation of *E1A* deletion in the vectors. To avoid targeting HEK293-encoded E1A during vector amplification, Cas9 was placed under the control of a tetracycline-regulatable CMV promoter, and vectors were amplified in T-REx-293 cells, a derivative of HEK293 cells stably expressing the tetracycline repressor. Western blot analysis confirmed the expression of Cas9 (Figure S1). The gRNA sequences were transcribed from the human U6 promoter (Figure 2).

Cas9 cleavage reporter assays reveal functional E1A-targeting gRNAs

To evaluate the functionality of the gRNAs, DNA from HeLa cells transduced with Cas9-gRNA expression vectors and infected with HAdV-5 was subjected to T7 endonuclease mismatch assays. Briefly, PCR amplicons of the region spanning the target sites were denatured, reannealed, and treated with T7 endonuclease to allow cleavage of mismatches arising after annealing of altered and unaltered target sites. Agarose gel electrophoresis identified cleavage products for gRNAs 1, 2, 7, 8, and 9 (Figure 3A), indicating that these gRNAs were capable of cleaving their targets. The functionality of two of these gRNAs selected for further experiments (gRNAs 8 and 9) was validated in a surrogate reporter system based on expression of an RFP-EGFP fusion protein.[38] Briefly, we inserted the *E1A* target sequences between an RFP-encoding sequence and two out-of-frame EGFP-encoding sequences, giving rise to a fusion protein that was only active for RFP but not for EGFP (Figure 3B). Upon insertion of indels into the target site by CRISPR-Cas9, the generated frameshift mutations were expected to render EGFP in frame with RFP, resulting in red and green fluorescence. Microscopy revealed the appearance of such cells in the presence of the Cas9/gRNA vectors (Figure 3C), indicating the functionality of the gRNAs. No green fluorescent cells appeared upon treatment with vectors expressing a non-targeting gRNA or Cas9 alone.

CRISPR-Cas9 decreases the generation of infectious viral particles and viral DNA

To test whether gRNAs 8 and 9 were capable of inhibiting replication of HAdV-5, we transduced HeLa cells with the respective adenoviral vectors or negative control vectors and infected them with HAdV-5 24 h after transduction. Two days after the infection, the number of infectious virus particles was determined. As shown in Figure 4A, gRNAs 8 and 9 decreased infectious virus particle numbers by one order of magnitude (90.4%) and 1.9 orders of magnitude (98.8%), respectively, compared with the non-targeting control gRNA. To examine whether inhibition can be maintained over a longer period of time, we conducted infection experiments over a period of 6 days and observed a decrease in the number of infectious virus progeny at all time points. At the latest time point, infectious HAdV-5 virus progeny was reduced by 0.6 orders of magnitude (75%) and 0.7 orders of magnitude (80%), respectively, compared with the non-targeting gRNA. CRISPR-Cas9 also decreased viral genome copy numbers; viral DNA was reduced by 0.87 orders of magnitude (86.5%) and 1.6 orders of magnitude (97.6%) by gRNAs 8 and 9, respectively. Because E1A activity was obviously not completely

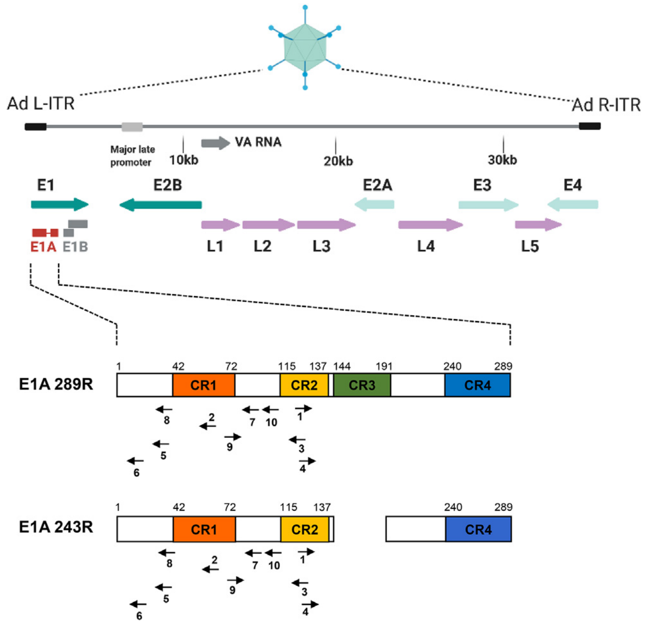

Figure 1. Binding sites of E1A-targeting gRNAs

The E1A gene serving as the target for CRISPR-Cas9-mediated cleavage of the viral DNA is shown in detail. The gRNAs binding to E1A are indicated by arrows numbered 1–10. The orientation of the arrows indicates binding to the plus and minus strand, respectively. The binding sites within the two most important E1A isoforms (243R and 289R) are depicted.

abrogated by CRISPR-Cas9, the otherwise replication-deficient, *E1A*-lacking vectors were also replicated in those cells (Figure S2). The amplification of the vectors was highest in the presence of the non-targeting gRNA, while it was reduced when E1A-targeting gRNAs were expressed.

A combination of CRISPR-Cas9 and CDV leads to increased inhibition of virus replication

Because we expected high target DNA numbers to represent a factor that would limit the degree of inhibition by CRISPR-Cas9 and potentially enhance the CRISPR-Cas9-mediated effect, we additionally treated the cells for 6 days with the viral DNA synthesis-inhibiting nucleoside analog CDV at concentrations of 10 and 30 μM. CDV at 30 μM reflects *in vivo* peak serum levels typically achieved after intravenous administration of CDV.[39] The CRISPR-Cas9 approach alone decreased the number of infectious virus particles by approximately one order of magnitude at all time points (Figure 5A). On day 2 post infection, the higher CDV concentration of 30 μM led to a comparable reduction in the number of infectious virus particles. However, at later time points, treatment with 30 μM CDV decreased the output of infectious virus particles more efficiently than CRISPR-Cas9. The combination did not have an additive effect at this CDV concentration, probably because of the already very high inhibitory effect exerted by CDV alone. However, at the lower concentration of 10 μM, CDV alone showed a similar degree of inhibition compared with CRISPR-Cas9, and a combination of both led to a pronounced

A

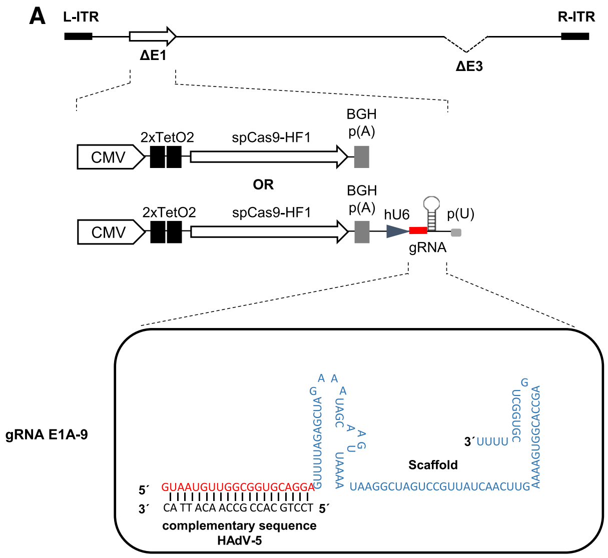

B

gRNA name	Target sequence 5´-3´	Target site in HAdV-5 (AY339865.1)
E1A #1	GATCGATCTTACCTGCCACG	916-935
E1A #2	GCCTCCTCGTTGGGATCTTC	722-741
E1A #3	GATCGATCACCTCCGGTACA	904-923
E1A #4	GATCTTACCTGCCACGAGGC	920-939
E1A #5	GGTTCAAAATGGCTAGGAGG	659-678
E1A #6	GATCAGCTGGTCCAAAAGAC	612-631
E1A #7	GTGAGGCGGCTCCGGAGAAC	822-841
E1A #8	GGTGGTTCAAAATGGCTAGG	662-681
E1A #9	GTAATGTTGGCGGTGCAGGA	767-786
E1A #10	GCTGCTCGGGCTGCCGGGAA	844-863
Non-targeting (NT)	GGAGCGATGATACGCGGTGC	

Figure 2. Structure of the recombinant adenovirus vector constructs and gRNA sequences incorporated into them

(A) Schematic of HAdV-5-based vectors with inserted individual gRNA sequences. All adenoviral vectors are based on the HAdV-5-derived vector pAd/PL-DEST (Thermo Fisher Scientific) and lack the E1 and E3 regions. Cas9 and gRNA expression cassettes were inserted into the deleted E1 region. The expression of spCas9-HF1 is driven by a tetracycline repressor-controlled CMV promoter comprising two binding sites for the repressor (2×TetO2). The expression of the individual targeting or non-targeting gRNAs is under control of a constitutive human U6 (hU6) promoter. The structure and sequence of the gRNAs are exemplarily shown for E1A gRNA 9 bound to its target site. The control vector containing only the Cas9 expression cassette is also depicted. (B) Target sequences for the individual gRNAs and their positions within the HAdV-5 genome (AY339865.1).

additive effect at all later time points. At the latest time point, the combination of CRISPR-Cas9 and CDV resulted in an additive effect of 2.76 and 2.97 orders of magnitude in comparison with the sole inhibitory effects of CRISPR-Cas9 and CDV, respectively (Figure 5B), culminating in a total reduction of infectious virus particles by 3.47 orders of magnitude (99.97%) compared with treatment with the non-targeting gRNA in the absence of CDV.

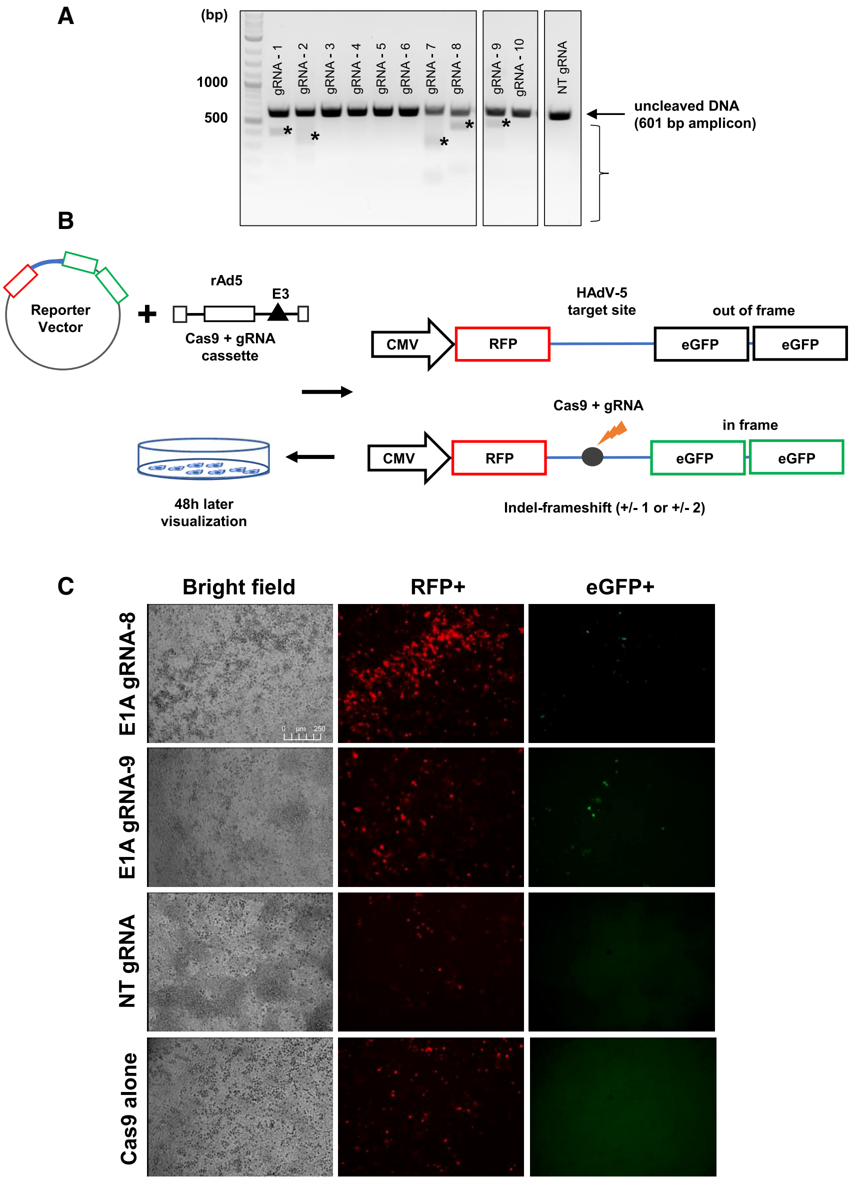

Figure 3. Detection of CRISPR-Cas9-mediated gene editing in T7 endonuclease mismatch assays and surrogate reporter assays

(A) HeLa cells were transduced with the adenoviral CRISPR-Cas9 expression vectors containing either Cas9 alone or Cas9 in combination with the individual targeting gRNAs or a non-targeting (NT) gRNA at an MOI of 100, followed by infection with HAdV-5 at an MOI of 0.01 24 h later. Four days post infection, DNA was isolated, and the target region comprising all target sites was amplified by PCR. The amplicon DNA was heat denatured, re-annealed to form heteroduplex DNA, subjected to T7 endonuclease I treatment, and analyzed by agarose gel electrophoresis. The uncleaved DNA band is indicated with an arrow. Cleavage products indicating heteroduplex DNA formed because of insertion of mutations by CRISPR-Cas9 at the specific target sites are indicated with asterisks. (B) Schematic of the EGFP surrogate reporter vectors and of the methodology. A fusion protein of RFP (red) and two out-of-frame EGFP copies (black), each in a different reading frame, are expressed from a CMV promoter. The E1A region comprising the individual target sites was inserted into the linker region between the RFP and EGFP sequences. Indel formation as a consequence of the repair of DNA double-strand breaks generated by CRISPR-Cas9 leads to frameshifts and expression of functional EGFP (green), indicating gRNA functionality. RFP expression (red) from the same vectors serves as a transfection control. (C) HeLa cells were transfected with the reporter vector and transduced with one of the recombinant adenoviral vectors expressing Cas9 alone or Cas9 in combination with a targeting or NT gRNA. Fluorescence was monitored 48 h post transduction with a Leica DMi8 System. Bright-field and red and green fluorescence images at a magnification of 10× are shown. Microscopy settings were as follows: HC PL FLUOTAR CS 10×/0.40 DRY; Camera Leica DFC 360FX: active resolution, 1,392 × 1,040; pixel bit depth, 12/8 bit; pixel size, 6.45 × 6.45 μm; live image with 1,392 × 1,040 at 20 images/s.

A combination of four gRNAs enhances inhibition of virus replication

Targeting a virus with more than one gRNA is not only beneficial for the overall inhibitory effect, it is also mandatory because introduction of indels that do not change the reading frame by a single gRNA would inevitably generate escape mutants that could no longer be targeted by the initial gRNA, consequently rendering them resistant. Thus, the expression of four gRNAs was combined. We combined the previously evaluated gRNAs 8 and 9 with gRNAs 1 and 7. Similar to gRNAs 8 and 9, the functionality of gRNAs 1 and 7 was in addition to the assessment in T7 endonuclease assays, which had indicated functionality (Figure 3A) also proven in the EGFP reporter system (Figure S3). The four gRNAs were expressed in four cassettes, each containing an individual RNA polymerase III promoter (Figure 6A). A control vector expressing four identical non-targeting gRNAs was also generated. The capacity of the 4-gRNA vector to inhibit HAdV-5 replication was assessed over a period of 6 days (Figure 6B). The degree of inhibition was significantly more pronounced than that achieved with vectors containing only one gRNA. While the virus concentration in cells treated with the non-targeting gRNA construct increased to between 1e+05 and 1e+06 infectious particles/mL, it remained around 1e+02–1e+03 infectious particles/mL in cells treated with the targeting gRNAs construct. At the latest time point, *E1A* targeting reduced infectious virus progeny by 2.8 orders of magnitude (99.8%) compared with the non-targeting control.

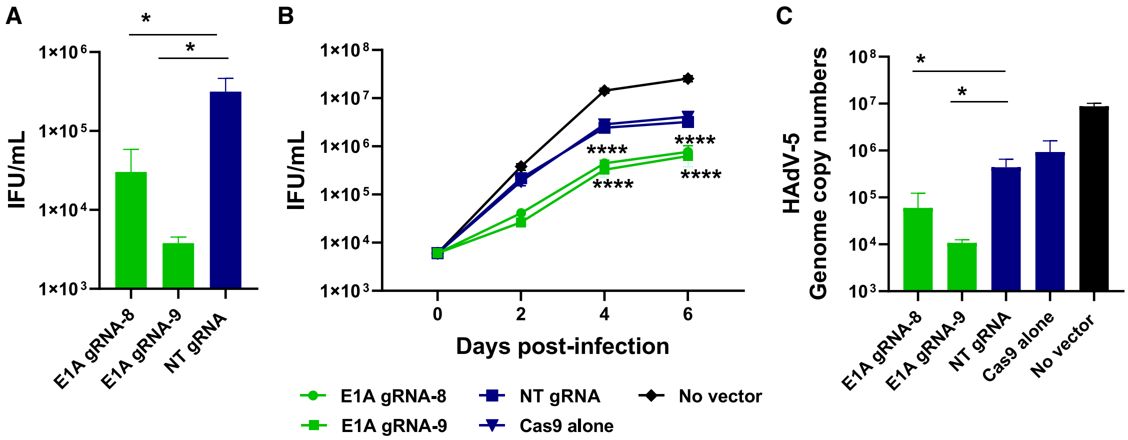

Figure 4. E1A-targeting gRNAs significantly decrease the numbers of infectious viral particles and HAdV-5 genome copy numbers

(A) HeLa cells were transduced with adenoviral vectors containing either Cas9 alone or Cas9 in combination with targeting gRNAs 8 or 9 or with an NT gRNA at an MOI of 100. 24 h after transduction, cells were infected with HAdV-5 at an MOI of 0.01. Numbers of infectious virus particles were determined on day 2 post infection and were expressed as infectious units (IFUs) per milliliter. Data represent the means (n = 3) ± SD of triplicate infections of a representative experiment of 3. $^{*}p < 0.05$. (B) HeLa cells were transduced and infected as in (A) and grown for a prolonged period of time. Numbers of infectious virus particles were determined at time points 0, 2, 4, and 6 days post infection. Data represent the means (n = 3) ± SD of triplicate infections of a representative experiment of 3. $^{****}p < 0.0001$. (C) Same experimental setup as in (A) with the difference that HAdV-5 genome copy numbers were determined by qPCR. Data represent the means (n = 3) ± SD of triplicate infections of a representative experiment of 3. $^{*}p < 0.05$.

To test whether this vector was also potent enough to inhibit HAdV-5 replication when applied shortly after infection, HeLa cells were first infected with HAdV-5, followed by transduction with the vectors 6 h post infection. Because secondary infection events taking place in the cultures at later time points would occur in the presence of already established high levels of Cas9 and gRNAs, no longer representing conditions where Cas9 and gRNA levels have to be built up first, infectious virus particle numbers were only determined 48 h post infection. Indeed, inhibition of HAdV-5 replication was also strong under these conditions (Figure 6C), suggesting that the buildup of sufficiently high levels of CRISPR effectors must be rapid. The inhibition of HAdV-5 replication by CRISPR-Cas9 was in general not restricted to HeLa cells but occurred in other cells, such as A549 cells, as well (Figure S4). Moreover, this was also observed when we increased the number of viral targets relative to vector copy numbers by raising the MOI for HAdV-5 to up to 50 to generate a more realistic scenario in which one vector molecule encounters one wild-type virus per cell (1:1 ratio) (Figure S5).

Because the 4-gRNA expression cassette is relatively large, and given the fact that the cloning space in certain delivery vectors (e.g., AAV vectors) is limited, we thought to generate an alternative, space-saving expression cassette (Figure 7A). In this cassette, the four targeting or non-targeting gRNAs are separated by artificial, non-targeting, pri-microRNA (miRNA) sequences based on the murine mmu-miR-155 pri-miRNA scaffold[40] and are transcribed as a polycistronic gRNA-miRNA precursor from a single RNA polymerase III promoter. Individual gRNAs are liberated from this precursor by DROSHA, which cleaves pri-miRNAs at a specific site at the base of each hairpin.[41,42] The exact DROSHA cleavage site for mmu-miR-155 has been determined previously, and the mmu-miR-155 scaffold has been developed into an amiRNA expression system.[43] Approaches for multiplex expression of gRNAs from polycistronic gRNA-amiRNA precursors have been proven to be functional in other contexts.[44,45] The final constructs were tested for their ability to inhibit HAdV-5 replication as before, and the two different vector types for multiplex gRNA expression were also evaluated side by side; a comparison of inhibition of HAdV-5 replication by vectors containing four separate expression cassettes, as depicted in Figure 6B, with that by vectors expressing the gRNAs from a common promoter that was tested in parallel is shown in Figure 7B. Over a period of 6 days, the degree of inhibition exerted by the two vector types was very similar. 6 days post infection, the vector expressing the gRNAs from a single promoter also drastically reduced the output of infectious virus progeny (by three orders of magnitude), and it was also able to decrease the number of infectious virus particles when applied to already infected HeLa cells 6 h post infection (Figure 7C).

Next-generation sequencing-based analysis of CRISPR-Cas9-induced mutations

To confirm the introduction of mutations into *E1A*, DNA was isolated from HAdV-5 infected cells that had been transduced with the adenoviral CRISPR-Cas9 vector expressing the four gRNAs from individual promoters or from cells that had only been infected with HAdV-5 (background control). The target region was amplified by PCR, and the amplified DNA was sequenced. Of 1,179 reads mapped to the amplicon sequence, 83.89% harbored deletions, insertions, or inversions (Figure 8A). When combining two or more gRNAs, generation of deletions between target sites is expected to

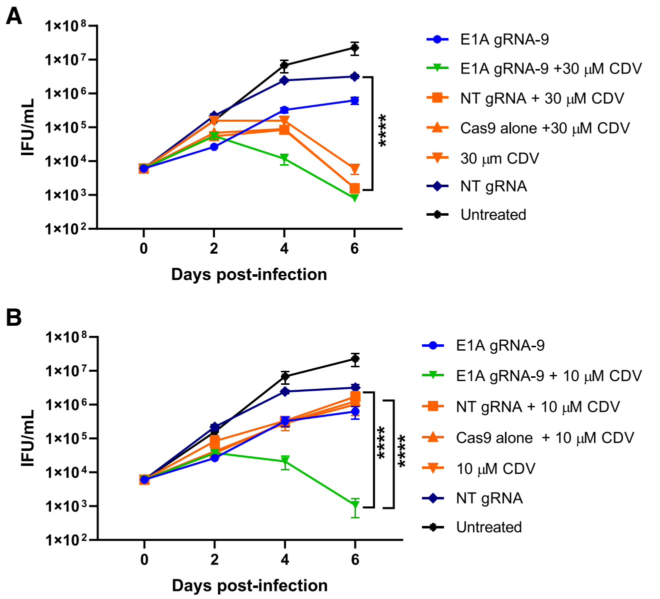

Figure 5. Low doses of CDV increase inhibition of HAdV-5 replication by CRISPR-Cas9

(A and B) HeLa cells were transduced with the adenoviral CRISPR-Cas9 expression vectors containing either Cas9 alone or Cas9 in combination with E1A gRNA 9 or an NT gRNA at an MOI of 100, followed by infection with HAdV-5 at an MOI of 0.01 24 h later. Concomitant with infection, the cultures were treated with or without CDV at a concentration of 30 μM (A) and 10 μM (B), respectively. Numbers of infectious HAdV-5 particles were determined on days 0, 2, 4, and 6 post infection and expressed as IFUs per milliliter. Data represent the means (n = 3) ± SD of triplicate infections of a representative experiment of 3. ****p < 0.0001.

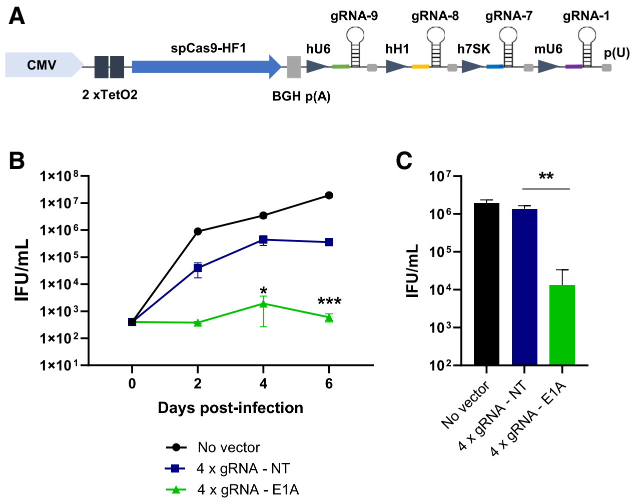

Figure 6. Targeting E1A with 4 different gRNAs potently inhibits HAdV-5 replication

(A) Schematic of the HAdV-5-based CRISPR-Cas9 vectors for multiplex gRNA expression. The sequences of gRNAs 1, 7, 8, and 9 were expressed from individual constitutive RNA polymerase III promoters (mU6, h7SK, hH1, and hU6). An analogous vector containing 4 identical NT gRNAs instead of the targeting gRNAs was constructed as well. SpCas9-HF1 was expressed from a doxycycline-regulatable CMV promoter harboring two TetO2 binding sites for the tetracycline repressor. (B) HeLa cells were transduced with the adenoviral vector containing Cas9 in combination with gRNAs 1, 7, 8, and 9 or with a control vector carrying 4 NTNT gRNAs at an MOI of 100, followed by infection of the cells with HAdV-5 at an MOI of 0.01 24 h later. Numbers of infectious virus particles were determined at the indicated time points and expressed as IFUs per milliliter. Data represent the means (n = 3) ± SD of triplicate infections of a representative experiment of 3. *p < 0.05, ***p < 0.001. (C) Experimental settings were as in (A) with the difference that HeLa cells were infected with HAdV-5 6 h prior to transduction with the vectors. Numbers of infectious virus particles were determined on day 2 post infection and expressed as IFUs per milliliter. Data represent the means (n = 3) ± SD of triplicate infections of a representative experiment of 3. **p < 0.01.

be the most frequent alteration. Indeed, sequencing indicated that deletions between target sites were the most dominant type of mutations. Small indels at the cleavage sites were almost negligible. We found deletions between all target sites, the most frequent of which were those between the target sites for gRNAs 9 and 1, while the least frequent were those between gRNA 8 and other target sites. All other deletions ranged in between. The region comprising the target site for gRNA 7 was most heavily affected by the mutations because it was either completely deleted (in 57.3% of all reads mapped to the amplicon region) or showed deletions going leftward or rightward toward the adjacent target sites. Figure 8B shows a multiple-sequence alignment of mutated sequences mapped to this region. Additionally, but to a lesser extent, we identified inversions between target sites and duplications in some cases coupled with deletions. The identified mutations are shown in Figure 8C. Almost all deletions and inversions led to frameshifts and, consequently, to truncated E1A proteins lacking at least CR2, CR3, and CR4 and, in most cases, most of the linker between CR1 and CR2 (Figure 8D). For some of the mutations depicted in Figure 8C, a few more variants existed that differed in harboring or lacking an additional single nucleotide at the cleavage site of gRNA 9 in certain types of junctions. Importantly, however, all of these variants showed frame shifts starting at the cleavage site of gRNA 9 and resulted in truncated proteins with out-of-frame sequences from amino acids 74 or 75 onward. This pattern occurred in all of these cases regardless of which other types of mutations in regions farther downstream had additionally taken place. We found one case in which specific joining of the fragments caused the amino acid sequence to jump back into the original reading frame at a position farther downstream (indicated as del 9-7 in Figure 8C). This type of joining is caused by offset cleavage by 1 nt or, more likely, removal of an end-standing nucleotide prior to repair of the gap. However, this specific mutation constituted less than 1% of all deletions, inversions, and duplications in which gRNA 9 was involved. In general, we observed cleavage only at unique positions. No small indels or larger deletions, insertions, or duplications were found in DNA isolated from cells infected with HAdV-5 in the absence of Cas9. The data indicated that ∼16% of E1A sequences represented wild-type E1A, likely originating from viruses that had not been reached by CRISPR-Cas9. Accordingly, virus recovered after targeting was able to replicate and still amenable to CRISPR-Cas9-mediated

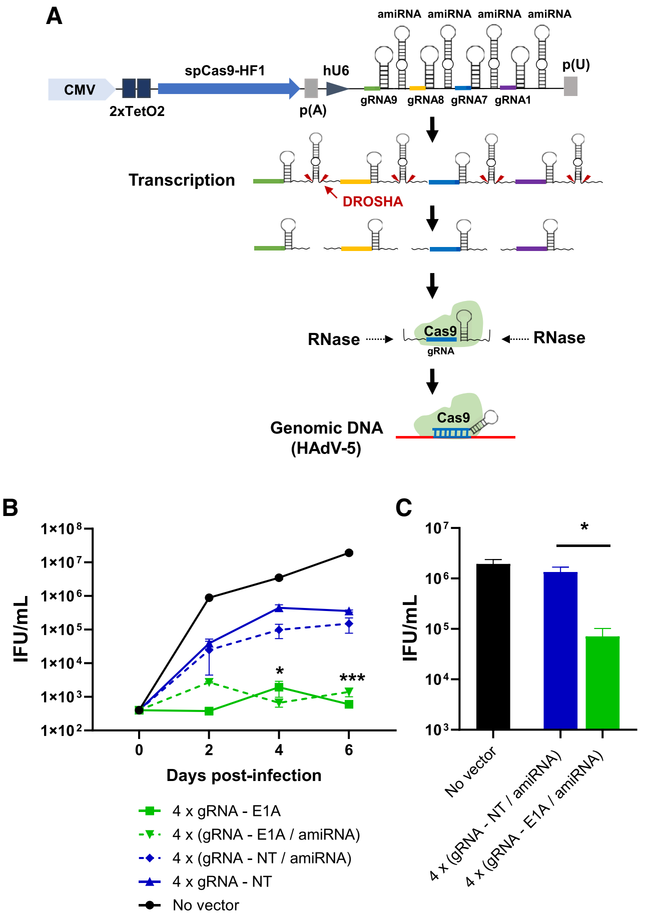

Figure 7. Concatemerized gRNAs separated by NT amiRNA spacers allow potent inhibition of HAdV-5 replication

(A) Schematic of the adenoviral vector for multiplex expression of concatemerized gRNAs and processing of the primary transcripts within the cell. SpCas9-HF1 expression is under control of a doxycycline-regulatable CMV promoter harboring two TetO2 binding sites for the tetracycline repressor, and multiplexed gRNAs separated by NT amiRNA are expressed from a common hU6 promoter. A negative control vector carrying 4 NT gRNAs was constructed in an analogous manner. The individual gRNAs are released from the primary transcript by DROSHA-mediated cleavage at the base of each interjacent amiRNA hairpin. The gRNA intermediates are further trimmed by Cas9 to give rise to the mature gRNAs. (B) HeLa cells were transduced with the adenoviral vectors containing Cas9 in combination with the concatemerized targeting gRNAs 1, 7, 8, and 9 or with a control vector bearing Cas9 in combination with 4 NT gRNAs at an MOI of 100. 24 h after transduction, the cells were infected with HAdV-5 at an MOI of 0.01. Numbers of infectious virus particles were determined at the indicated time points and expressed as IFUs per milliliter. For better comparison, the values for the vectors expressing the 4 gRNAs from individual promoters and those for the only-HAdV-5 control presented in Figure 6D are also shown here because the respective transfection/infection experiments were conducted side by side. Data represent the means (n = 3) ± SD of triplicate infections of a representative experiment of 3. *$p < 0.05$, ***$p < 0.001$. (C) Experimental settings were as in (B) with the difference that HeLa cells were infected with HAdV-5 6 h prior to transduction with the vectors. Numbers of infectious virus particles were determined on day 2 post infection and expressed as IFUs per milliliter. Data represent the means (n = 3) ± SD of triplicate infections of a representative experiment of 3. *$p < 0.05$.

inhibition in a subsequent round of targeting (Figure S6). An evaluation of the specificity of SpCas9-HF1 targeting did not reveal cleavage at sites with some potential for off-target cleavage (Figure S7).

DISCUSSION

Our data indicated that replicating adenoviral DNA is accessible to CRISPR-Cas9 because targeting of *E1A* can lead to a decrease in infectious virus progeny. Moreover, the time window during which CRISPR-Cas9 effectors have access to the viral DNA before it is encapsidated seems to be large enough to allow effective cleavage. The rationale for choosing *E1A* as a target was based on our previous data, which revealed that RNAi-mediated reduction of only certain early viral gene products that directly (pTP, viral DNA polymerase) or indirectly (E1A) affect viral DNA replication leads to a decrease in infectious virus progeny.[46] Preventing the accumulation of high numbers of target molecules that would occur when viral DNA replication started seems to have been the key to these experiments and is probably also required in CRISPR-Cas9-based approaches. We expected particularly *E1A* to be a promising target because it is required for expression of other adenoviral genes, and its knockout would consequently render mutant viruses barely active. In contrast, pTP or DNA polymerase mutants would also be replication deficient but still produce functional E1A proteins with multiple consequences for various cellular and viral processes.

Cleavage of the target DNA obviously occurred soon enough after infection to exert a negative effect on viral replication. This effect may be due to impairment of E1A-mediated transcriptional activation of other viral genes. We observed previously that knockdown of *E1A* expression by RNAi had an immediate negative effect on expression of later viral genes involved in viral DNA replication and, consequently, on viral DNA copy numbers.[46] Knockout of E1A by CRISPR-Cas9 probably caused the same effect. In addition, it is likely that cleavage of viral DNA directly reduced viral genome copy numbers and hindered synthesis of full-length viral DNA. Together, these effects probably ensured a reasonable ratio between the target molecules and CRISPR-Cas9 effectors. Treatment with low concentrations of CDV seemed to have shifted this ratio further in favor of CRISPR-Cas9 effectors, leading to even higher degrees of inhibition (Figure 5B). The time window during which viral DNA is accessible becomes even smaller when trying to target infected cells. However, the accumulation of gRNA and Cas9 effectors seemed to have occurred quickly enough to efficiently cleave the target DNA, even in those cells (Figures 6C and 7C). It is possible that the replication of the adenoviral vectors, which was triggered by HAdV-5 at least to some extent even in the presence of *E1A*-targeting gRNAs (Figure S2), contributed to building up sufficiently high levels in a short time. We have also proven this beneficial amplification effect when targeting HAdV-5 mRNA with adenoviral vector-expressed artificial miRNAs.[47] Such systems are self-balancing insofar as the vector will be generated as long as inhibition of wild-type virus activity is not complete, which may generate a supply of the vector at sites of infection *in vivo*. Adenoviral vectors and, to an even higher extent,

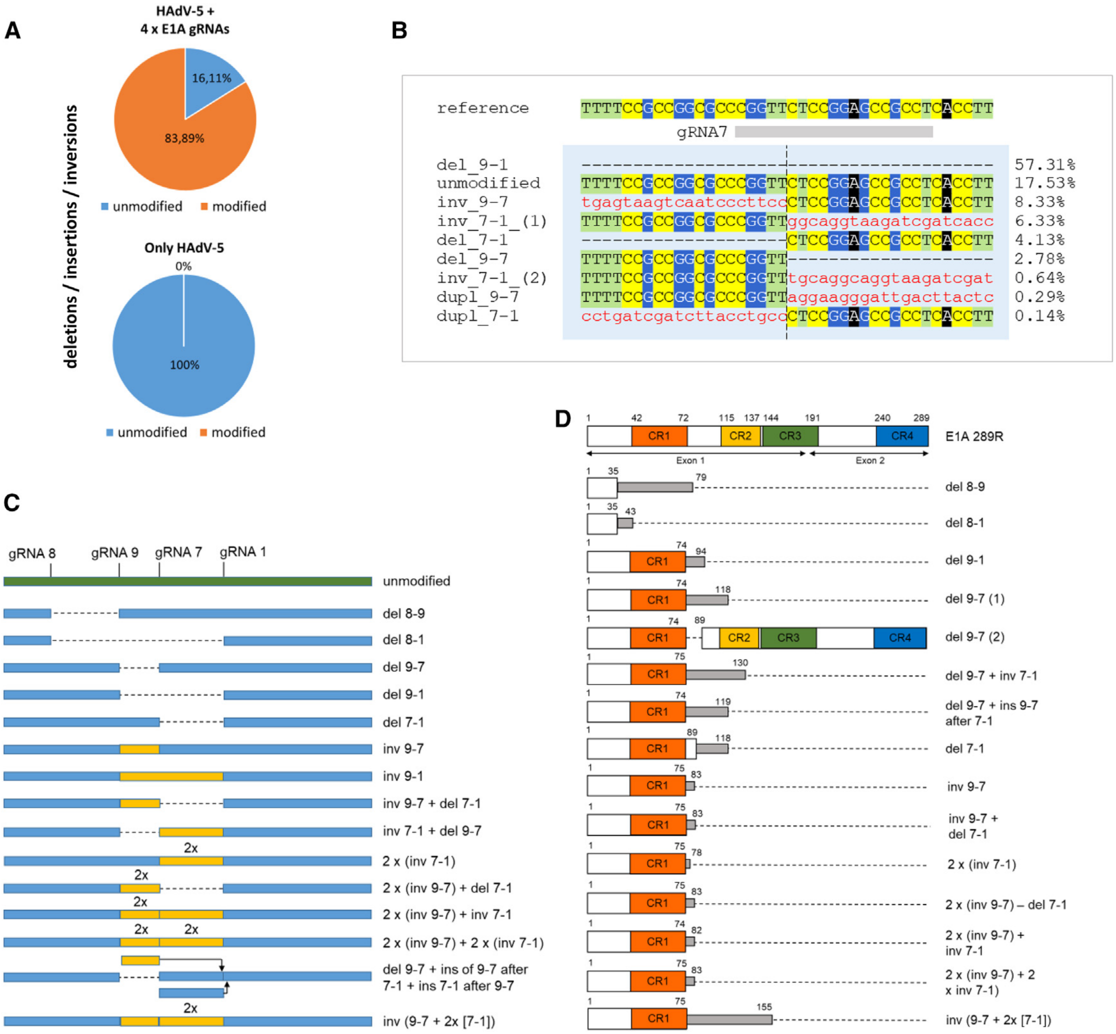

Figure 8. Targeting of E1A with 4 different gRNAs primarily leads to deletions, inversions, and duplications in E1A

HeLa cells were transduced with adenoviral vectors harboring Cas9 in combination with the 4 gRNAs 1, 7, 8, and 9 expressed from separate promoters at an MOI of 100 or were mock transduced. 24 h after transduction, the cells were infected with HAdV-5 at an MOI of 0.01. Two days post infection, DNA was isolated from the cells, a large region comprising all E1A target sites was amplified by PCR, and the amplicon DNA was subjected to loop sequencing. (A) Total percentage of deletions/insertions/inversions in the target region. (B) Alignment of reads around the most heavily affected region comprising the target site for gRNA 7. Deletions (del; dashed lines), inversions (inv; in red and lowercase), and duplications (dupl; in red and lowercase) are shown. The cleavage position for gRNA 7 is indicated with a vertical dashed line. The frequency of the mutations (percentage of reads mapping to the target region) are given on the right of the alignments. (C) Types of deletions, inversions, and duplications detectable in the target region. Deletions are indicated with dashed lines, and inversions are colored in yellow. Changed positions of fragments are indicated with arrows. (D) Consequences of the mutations for the E1A reading frame. Narrow boxes in gray symbolize the length of the protein. Larger open boxes represent the open reading frames. Conserved regions (CRs) within the open reading frames are colored. Numbers above the individual schemes indicate the amino acid positions where the proteins and the open reading frames start and end, respectively. The start and endpoints of the CRs are given for comparison.

adeno-associated virus (AAV) vectors,[48] are among the most promising viral vectors for gene delivery *in vivo* and are conceivable delivery vehicles for any CRISPR-Cas9-based application. AAV vectors are also amplified in the presence of wild-type adenoviruses.[49] However, the use of AAV vectors is often hampered by their limited cloning capacity, which prevents insertion of larger fragments. This also applies to the multi-gRNA expression cassettes, in which each gRNA is expressed from individual promoters. The fact that our much shorter, polycistronic gRNA/pri-amiRNA expression cassette performed equally well opens up the possibility of its incorporation into AAV vectors to permit efficient *in vivo* delivery and amplification in adenovirus-infected cells. Moreover, this design allows incorporation of a higher number of gRNAs in a space-saving manner to simultaneously target a wider range of adenovirus serotypes such as—just to mention one conceivable application—the whole spectrum of serotypes associated with ocular infections.

Targeting of *E1A* with four different gRNAs caused a variety of deletions, inversions, or duplications (Figure 8), of which the majority (>99%) caused frameshifts rendering E1A defective in functions residing in CR2, CR3, CR4, and, when the target site for gRNA8 was involved, CR1. There are numerous consequences for E1A function. To give one example, the lack of CR3, which is important for transactivation of other early adenoviral promoters,[50,51] is expected to result in a strong inhibitory effect on viral DNA replication because the expression of viral E2 genes encoding the proteins for viral DNA synthesis is dependent on transactivation mediated by CR3. E1A functions residing in other CRs affected by the mutations are extremely heterogeneous and affect processes such as further activation of viral and cellular transcription, de-repression of transcription, cell cycle entry control, differentiation, stabilization of p53, immortalization of cells, apoptosis, or proteasomal degradation,[30,31] all of which contribute to viral replication.

E1A exerts most of its functions in the nucleus,[31] and lack of its import into the nucleus has severe consequences for E1A function.[52,53] All frameshift mutations removed the canonical nuclear localization signal (NLS) at the C terminus of E1A (amino acids [aa] 258–263 and 285–288), whose deletion impairs nuclear localization,[54–56] and a second noncanonical NLS located within CR3 (aa 142–182).[57,58] A sequence in the N-terminal/CR1 part of E1A (aa 30–69) that seems to contribute to the nuclear localization of E1A, albeit to a lesser extent,[57] was only removed by the largest deletions involving cleavage by gRNA8. Thus, the observed mutations probably had a more general impact by negatively affecting the subcellular localization of E1A, with consequences for its activity, regardless of which other functions of the protein had been directly eliminated by the mutations. Together, the loss of functions directly associated with the mutated regions and the impairment of the proper localization of the E1A remnants may explain the pronounced inhibitory effect on virus multiplication.

The mutation frequency of approximately 84% translated into a decrease of 2.5–2.8 orders of magnitude in infectious virus progeny. This is about the same degree of inhibition we had observed previously when targeting HAdV-5 with small interfering RNAs (siRNAs) or amiRNAs.[46,47] Because potent siRNAs and amiRNAs typically reduce target RNA levels by 80%–90%, and assuming, in a very simplistic way, that the respective protein levels become decreased to a similar extent, the degree of inhibition reported here reflects what we had observed previously when targeting adenoviral RNA. Thus, CRISPR-Cas9-based methods appear to be capable of decreasing infectious virus progeny *in vitro* to approximately the same extent as RNAi-based methods. Because amiRNA-based methods have been shown to also inhibit adenovirus replication *in vivo*,[59] CRISPR-Cas9 may have similar potential. It was shown that AAV vector-mediated amiRNA delivery inhibited HAdV-5 replication not only locally in the main targeted organ (the liver) but led to a general decrease of virus load in the animals,[59] demonstrating that delivering anti-adenoviral effectors to the liver, which functions as a virus multiplicator, is a promising strategy to decrease the overall virus load in systemically infected animals. Analogously, by applying similar delivery strategies, CRISPR-Cas9 may have a chance to be developed into a therapeutic tool to treat disseminated adenovirus infections. Treatment would primarily involve targeting members of adenovirus species C (including HAdV-5), which frequently predominate in disseminated adenovirus infection, followed by members of species A and B.[21]

However, because of risks associated with any systemic delivery, topical treatment of localized infections would probably have a higher chance of realization. The eye constitutes an ideal gene therapy target. High vector titers can be achieved with small volumes, and because it is a largely closed organ, systemic distribution and side effects are minimized.[60,61] Accordingly, localized adenovirus infections of the eye (caused by adenoviruses of species D serotypes 8, 37, and 64 [previously classified as 19a] and, more recently, by serotypes 22, 54, 56, 82, and 85; species G serotypes 3, 7, and 11; and species E serotype 4)[62,63] can conceivably be treated topically with future therapeutics based on CRISPR-Cas9.

MATERIALS AND METHODS

Cell lines and viruses

HEK293 (human embryonic kidney, ATCC CRL-1573), T-REx-293 (stable integration of the tetracycline repressor gene, Thermo Fisher Scientific, R71007), HeLa (human epithelial carcinoma, ATCC CCL-2), and A549 (human epithelial lung carcinoma, ATCC CCL-185) cells were cultivated in Dulbecco's modified Eagle's medium (DMEM) with stabilized glutamine (Thermo Fisher Scientific, Vienna, Austria) supplemented with 10% fetal bovine serum (FBS; Thermo Fisher Scientific) in a humidified 5% CO_2 atmosphere at 37°C.

HAdV-5 (ATCC VR-5) was amplified in HEK293 cells; recombinant adenoviral vectors were amplified in T-REx-293 cells in the absence of doxycycline. Wild-type virus and recombinant vectors were purified by CsCl centrifugation or with a Fast Trap Adenovirus Purification and Concentration Kit (Merck/Millipore, Vienna, Austria). Titers of HAdV-5 were determined on HeLa cells with an Adeno-X Rapid Titer Kit according to the instructions of the manufacturer (Takara Bio Europe, Paris, France); titers of recombinant HAdV-5 were analogously determined on T-REx-293 cells in the absence of doxycycline.

CRISPR-Cas9 expression vectors

Vector designs and sequence analyses were performed in CLC Main Workbench 8.1.2. (QIAGEN, Hilden, Germany). To construct the individual Cas9/gRNA expression vectors, the SpCas9-HF1 coding sequence[37] was amplified by PCR from plasmid VP12 (obtained from Addgene) with primers Cas9 HF1 FW and Cas9 HF1 RV, and the fragment was cloned into the *Eco*RI and *Not*I sites of pENTR4 (Thermo Fisher Scientific). The tetracycline-regulatable CMV-tetO2 promoter was amplified by PCR from pcDNA6.2-GW/EmGFP-miR-luc (Thermo Fisher Scientific) with primers CMV Tet02_FW and CMV Tet02_FW, and the resulting fragment was cloned into

the *Nco*I and *Eco*RI sites of the same vector. For amplification and sequencing of the pENTR4-based vector, primers pENTR4 FW and RV were used.

Selection of gRNAs (Figure 2B) and estimation of targeting probabilities were performed with CHOP-CHOP,[64] CasFinder (2017, available online at http://arep.med.harvard.edu/CasFinder/), and CRISPOR (2020, available online at http://crispor.tefor.net/). The gRNAs were designed as described previously[65] to minimize off-target effects. Linear DNA fragments (gBLOCKS) containing individual targeting or non-targeting gRNAs under control of a human U6 promoter[66] were generated by gene synthesis (Integrated DNA Technologies, Coralville, IA, USA), and the individual expression cassettes were inserted into the *Hind*II and *NotI* sites of the SpCas9-HF1 expression cassette-containing intermediate vector, giving rise to vectors pENTR-CMV-TetO2-spCas9-HF1-hU6-gRNA 1–10, containing a single targeting gRNA each, and to the control vectors containing non-targeting gRNAs (pENTR-CMV-TetO2-spCas9-HF1-hU6-NT gRNA) or containing only the spCas9-HF1 expression cassette (pENTR-CMV-TetO2-spCas9-HF1).

For construction of the plasmid vectors containing 4 targeting or non-targeting gRNAs, each expressed from its own promoter (4× gRNA-E1A, 4× gRNA-NT), the individual expression cassettes were generated by gene synthesis (Biomatik, Kitchener, ON, Canada), and the fragments were transferred into the *Hind*II and *Not*I sites of the CMV-TetO2-spCas9-HF1 backbone. Vectors 4× (gRNA-E1A/amiRNA) and 4× (gRNA-NT/amiRNA) containing an array of 4 targeting or non-targeting gRNAs separated by non-targeting amiRNA hairpin sequences (originating from pcDNA6.2-GW/EmGFP-miR-neg, Thermo Fisher Scientific) were constructed in an analogous way.

The entire expression cassettes present in the plasmid vectors were eventually moved into the deleted E1 region of the adenoviral vector pAd/PL-DEST (Thermo Fisher Scientific) by employing the Gateway system for site-specific recombination between sequences flanking the cassettes and the corresponding sequences located on the adenoviral vector. The resulting adenoviral vectors were named Ad E1A-gRNA 1–10 (containing the individual targeting gRNAs 1–10), Ad NT-gRNA (containing a non-targeting gRNA), Ad 4× gRNA-E1A and Ad 4× gRNA-NT (containing 4 targeting and 4 non-targeting gRNAs, respectively, expressed from individual promoters), Ad 4× (gRNA-E1A/amiRNA) and 4× (gRNA-NT/amiRNA) (containing arrays of 4 targeting and 4 non-targeting gRNAs, respectively), and Ad Cas9 (containing only spCas9-HF1).

Restriction enzymes and DNA-modifying enzymes were purchased from New England Biolabs, Frankfurt am Main, Germany). PCR reactions were performed with Quick-Load Taq 2X Master Mix (New England Biolabs) and Q5 High-Fidelity DNA Polymerase (New England Biolabs). Plasmid DNA was extracted with a QIAprep Mini or Midi Kit, and PCR products were purified with a QIAquick PCR Purification Kit. All kits for nucleic acid purification were acquired from QIAGEN. All primers are listed in Table S1.

T7 endonuclease mismatch assay

1.5e+04 HeLa cells seeded into the wells of a 96-well plate were transduced with adenoviral vectors expressing Cas9 and individual gRNAs at an MOI of 100, followed by infection with HAdV-5 at an MOI of 0.01 24 h post transduction. On day 4 after infection with HAdV-5, DNA was isolated, and three nested PCR reactions using a Q5 High-Fidelity DNA Polymerase PCR System (New England Biolabs) and primer pairs T7E1 E1A Set1–Set3 (Table S1) were performed. Amplified DNA was heatdenatured and allowed to re-anneal to form heteroduplexes between altered and unaltered single strands. The samples were subsequently purified with a QIAquick PCR Purification Kit (QIAGEN), digested with T7 Endonuclease 1 (New England Biolabs), and analyzed by agarose gel electrophoresis.

EGFP reporter system

To detect cells with nuclease-induced mutations and test the functionality of the gRNAs, a surrogate reporter system (Figure 3B) was adopted.[38] Briefly, double-stranded, linear oligonucleotides representing E1A target regions, each carrying clusters of 2–3 individual target sites in close proximity to each other, were inserted into the *Eco*R1 and *Bam*HI sites of vector RVO1 (PNA Bio, Newbury Park, CA, USA), giving rise to vectors RV01-1, -3, and -4 (carrying the target sites for gRNAs 1, 3, and 4, respectively); RV01-2 and -9 (carrying the target sites for gRNAs 2 and 9, respectively); RV01-5, -6, and -8 (carrying the target sites for gRNAs 5, 6, and 8, respectively); and RV01-7 and -10 (carrying the target sites for gRNAs 7 and 10, respectively). The final RV01-based vectors were sequenced using primers RV01 sequencing FW and RV (Table S1). The inserted target sequences are listed in Table S2. 2e+04 to 5e+04 HeLa cells were seeded into the wells of a 96-well plate and transfected with 100 ng of reporter vectors using Lipofectamine 2000 (Thermo Fisher Scientific), followed by transduction with the recombinant Cas9/gRNA-expressing vectors. 48 h post transduction, pictures were acquired with a Leica DMi8 system and analyzed with the microscope software platform LAS X Life Science (Leica, Wetzlar, Germany).

Western blotting

Proteins were separated by sodium dodecyl sulfate-polyacrylamide gel electrophoresis on Mini-Protean TGX Precast protein gels (Bio-Rad, Hercules, CA, USA) and transferred onto nitrocellulose membranes (Bio-Rad) using a Turbo transfer system (Bio-Rad). Membranes were blocked with 5% BSA T-BST (500 mM Tris HCl [pH 7.5], 1.5 M NaCl, 0.05% Tween 20). SpCas9-HF1 and β-actin were detected with antibodies 7A9-3A3 (Cell Signaling Technology, Danvers, MA, USA) and GTX629630-25 (GeneTex, Irvine, CA, USA), respectively. Membranes were probed with the fluorescent secondary antibodies IRDye 800CW goat anti-mouse (925-32210, LI-COR Biosciences, Lincoln, NE, USA) and IRDye 680RD goat anti-rabbit (925-68071, LI-COR Biosciences), respectively, and bands were visualized with a ChemiDoc MP Imaging System (Bio-Rad).

Virus inhibition experiments

For prophylactic inhibition of virus replication, 1.5e+04 HeLa or A549 cells were seeded into the wells of a 96-well plate and transduced

with the recombinant adenoviruses at an MOI of 100. 24 h later, cells were infected with HAdV-5 at an MOI of 0.01. In an alternative approach, cells were first infected with HAdV-5 at an MOI of 0.01, followed by transduction with the adenoviral vectors 6 h after infection. For virus inhibition experiments at higher HAdV-5 MOIs, A549 cells were seeded into the wells of a 96-well plate as before and transduced with the recombinant adenoviruses at an MOI of 50, followed by infection with HAdV-5 24 h later at MOIs ranging from 0.05–50.

Determination of HAdV-5 and adenoviral vector DNA copy numbers

DNA was isolated from crude lysates with a QIAamp DNA Blood Mini Kit (QIAGEN), and HAdV-5 DNA was quantified by qPCR using a TaqMan primer/probe set (Table S1) specific for the adenoviral E3 gene. Adenovirus genome copy numbers were calculated by serial dilutions of an adenoviral reference DNA. qPCRs conditions were as follows: 1× (50°C for 30 s, 95°C for 3 min) and 40× (95°C for 10 s, 60°C for 30 s). Adenoviral vector DNA was quantified analogously with a primer/probe set specific for the Cas9-encoding part of the vectors.

Sequencing

For LoopSeq[67] adenoviral DNA isolated from cells transduced with recombinant adenoviruses and infected with HAdV-5 or only infected with HAdV-5 was subjected to PCR to amplify a segment in the left end of HAdV-5 containing all individual target sites. Amplification was performed with primers E1A/E1B Loop Seq FW and E1A/E1B Loop Seq RV (Table S1) and Q5 Hot Start High-Fidelity 2× Master Mix (New England Biolabs). The amplicon sequences were determined at Loop Genomics (San Jose, CA, USA). FASTQ data were analyzed via CRISPResso2[64] for quantification and location of specific CRISPR-Cas9-induced mutations. Potential off-target cleavage was analyzed by amplifying the respective regions with the primers specified in Table S1, and amplicon sequences were determined at Eurofins (Ebersberg, Germany). FASTQ data were analyzed with CRISPResso2.[68]

Statistical analysis

GraphPad Prism v.8.00 was used to analyze and graph the data. All data are expressed as mean ± standard deviation (SD). To test for statistical significance in the inhibition experiments, one-way or two-way ANOVA was used. For multiple comparisons, Dunnett's test was employed. Statistical significance is indicated for each experiment (*$p < 0.05$, **$p < 0.01$, ***$p < 0.001$).

DATA AVAILABILITY

The most relevant datasets generated and analyzed as part of this study are included in this published article and its supplemental information files. Raw data analyzed in the study are available from the corresponding author upon request.

SUPPLEMENTAL INFORMATION

Supplemental information can be found online at https://doi.org/10.1016/j.omtn.2023.02.033.

ACKNOWLEDGMENTS

This work was supported by the Austrian Science Fund through grant P 29976.

AUTHOR CONTRIBUTIONS

Z.D., F.R., A.W., A.J., K.Z., and R.K. were responsible for conception of the work and design of the experiments. Z.D., F.R., K.Z., A.J., I.K., and R.K. performed the experiments. A.W. and R.K. analyzed the data. Z.D., F.R., A.J., K.Z., and R.K. wrote the paper. A.W. and I.K. revised the work.

DECLARATION OF INTERESTS

The authors declare no competing interests.

REFERENCES

1. Chylinski, K., Makarova, K.S., Charpentier, E., and Koonin, E.V. (2014). Classification and evolution of type II CRISPR-Cas systems. Nucleic Acids Res. *42*, 6091–6105.
2. Doudna, J.A., and Charpentier, E. (2014). Genome editing. The new frontier of genome engineering with CRISPR-Cas9. Science *346*, 1258096.
3. Haeussler, M., and Concordet, J.P. (2016). Genome editing with CRISPR-cas9: can it get any better? J. Genet. Genom. *43*, 239–250.
4. Hsu, P.D., Lander, E.S., and Zhang, F. (2014). Development and applications of CRISPR-Cas9 for genome engineering. Cell *157*, 1262–1278.
5. de Buhr, H., and Lebbink, R.J. (2018). Harnessing CRISPR to combat human viral infections. Curr. Opin. Immunol. *54*, 123–129.
6. Lee, C. (2019). CRISPR/Cas9-Based antiviral strategy: current status and the potential challenge. Molecules *24*, 1349.
7. Siegrist, C.M., Kinahan, S.M., Settecerri, T., Greene, A.C., and Santarpia, J.L. (2020). CRISPR/Cas9 as an antiviral against Orthopoxviruses using an AAV vector. Sci. Rep. *10*, 19307.
8. Hübner, A., Petersen, B., Keil, G.M., Niemann, H., Mettenleiter, T.C., and Fuchs, W. (2018). Efficient inhibition of African swine fever virus replication by CRISPR/Cas9 targeting of the viral p30 gene (CP204L). Sci. Rep. *8*, 1449.
9. Chou, Y.Y., Krupp, A., Kaynor, C., Gaudin, R., Ma, M., Cahir-McFarland, E., and Kirchhausen, T. (2016). Inhibition of JCPyV infection mediated by targeted viral genome editing using CRISPR/Cas9. Sci. Rep. *6*, 36921.
10. Wollebo, H.S., Bellizzi, A., Kaminski, R., Hu, W., White, M.K., and Khalili, K. (2015). CRISPR/Cas9 system as an agent for eliminating polyomavirus JC infection. PLoS One *10*, e0136046.
11. Hagag, I.T., Wight, D.J., Bartsch, D., Sid, H., Jordan, I., Bertzbach, L.D., Schusser, B., and Kaufer, B.B. (2020). Abrogation of Marek's disease virus replication using CRISPR/Cas9. Sci. Rep. *10*, 10919.
12. Karimova, M., Beschorner, N., Dammermann, W., Chemnitz, J., Indenbirken, D., Bockmann, J.H., Grundhoff, A., Lüth, S., Buchholz, F., Schulze zur Wiesch, J., and Hauber, J. (2015). CRISPR/Cas9 nickase-mediated disruption of hepatitis B virus open reading frame S and X. Sci. Rep. *5*, 13734.
13. Oh, H.S., Neuhausser, W.M., Eggan, P., Angelova, M., Kirchner, R., Eggan, K.C., and Knipe, D.M. (2019). Herpesviral lytic gene functions render the viral genome susceptible to novel editing by CRISPR/Cas9. Elife *8*, e51662.
14. van Diemen, F.R., Kruse, E.M., Hooykaas, M.J.G., Bruggeling, C.E., Schürch, A.C., van Ham, P.M., Imhof, S.M., Nijhuis, M., Wiertz, E.J.H.J., and Lebbink, R.J. (2016). CRISPR/Cas9-Mediated genome editing of herpesviruses limits productive and latent infections. PLoS Pathog. *12*, e1005701.
15. Wu, B.W., Yee, M.B., Goldstein, R.S., and Kinchington, P.R. (2022). Antiviral targeting of varicella zoster virus replication and neuronal reactivation using CRISPR/Cas9 cleavage of the duplicated open reading frames 62/71. Viruses *14*. https://doi.org/10.3390/v14020378.

16. Gonçalves, M.A.F.V., and de Vries, A.A.F. (2006). Adenovirus: from foe to friend. Rev. Med. Virol. *16*, 167–186.

17. Robinson, C.M., Singh, G., Lee, J.Y., Dehghan, S., Rajaiya, J., Liu, E.B., Yousuf, M.A., Betensky, R.A., Jones, M.S., Dyer, D.W., et al. (2013). Molecular evolution of human adenoviruses. Sci. Rep. *3*, 1812.

18. Al-Heeti, O.M., Cathro, H.P., and Ison, M.G. (2022). Adenovirus infection and transplantation. Transplantation *106*, 920–927.

19. Echavarría, M. (2008). Adenoviruses in immunocompromised hosts. Clin. Microbiol. Rev. *21*, 704–715.

20. Kojaoghlanian, T., Flomenberg, P., and Horwitz, M.S. (2003). The impact of adenovirus infection on the immunocompromised host. Rev. Med. Virol. *13*, 155–171.

21. Lion, T. (2014). Adenovirus infections in immunocompetent and immunocompromised patients. Clin. Microbiol. Rev. *27*, 441–462.

22. Saha, B., and Parks, R.J. (2020). Recent advances in novel antiviral therapies against human adenovirus. Microorganisms *8*, 1284.

23. Vora, S.B., Brothers, A.W., and Englund, J.A. (2017). Renal toxicity in pediatric patients receiving cidofovir for the treatment of adenovirus infection. J. Pediatric Infect. Dis. Soc. *6*, 399–402.

24. Alvarez-Cardona, J.J., Whited, L.K., and Chemaly, R.F. (2020). Brincidofovir: understanding its unique profile and potential role against adenovirus and other viral infections. Future Microbiol. *15*, 389–400.

25. Hartline, C.B., Gustin, K.M., Wan, W.B., Ciesla, S.L., Beadle, J.R., Hostetler, K.Y., and Kern, E.R. (2005). Ether lipid-ester prodrugs of acyclic nucleoside phosphonates: activity against adenovirus replication in vitro. J. Infect. Dis. *191*, 396–399.

26. Paolino, K., Sande, J., Perez, E., Loechelt, B., Jantausch, B., Painter, W., Anderson, M., Tippin, T., Lanier, E.R., Fry, T., and DeBiasi, R.L. (2011). Eradication of disseminated adenovirus infection in a pediatric hematopoietic stem cell transplantation recipient using the novel antiviral agent CMX001. J. Clin. Virol. *50*, 167–170.

27. Berk, A.J. (2005). Recent lessons in gene expression, cell cycle control, and cell biology from adenovirus. Oncogene *24*, 7673–7685.

28. Costa, R., Akkerman, N., Graves, D., Crisostomo, L., Bachus, S., and Pelka, P. (2020). Characterization of adenovirus 5 E1A exon 1 deletion mutants in the viral replicative cycle. Viruses *12*, 213. https://doi.org/10.3390/v12020213.

29. Gallimore, P.H., and Turnell, A.S. (2001). Adenovirus E1A: remodelling the host cell, a life or death experience. Oncogene *20*, 7824–7835.

30. King, C.R., Zhang, A., Tessier, T.M., Gameiro, S.F., and Mymryk, J.S. (2018). Hacking the cell: network intrusion and exploitation by adenovirus E1A. mBio *9*, e00390-18. https://doi.org/10.1128/mBio.00390-18.

31. Pelka, P., Ablack, J.N.G., Fonseca, G.J., Yousef, A.F., and Mymryk, J.S. (2008). Intrinsic structural disorder in adenovirus E1A: a viral molecular hub linking multiple diverse processes. J. Virol. *82*, 7252–7263.

32. Pelka, P., Ablack, J.N.G., Shuen, M., Yousef, A.F., Rasti, M., Grand, R.J., Turnell, A.S., and Mymryk, J.S. (2009). Identification of a second independent binding site for the pCAF acetyltransferase in adenovirus E1A. Virology *391*, 90–98.

33. Lillie, J.W., Hai, T., Coukos, W.J., Lee, K.A., Martin, K.J., and Green, M.R. (1989). Transcriptional activation of adenoviral early genes. Curr. Top. Microbiol. Immunol. *144*, 191–195.

34. Avvakumov, N., Kajon, A.E., Hoeben, R.C., and Mymryk, J.S. (2004). Comprehensive sequence analysis of the E1A proteins of human and simian adenoviruses. Virology *329*, 477–492.

35. Arulsundaram, V.D., Webb, P., Yousef, A.F., Pelka, P., Fonseca, G.J., Baxter, J.D., Walfish, P.G., and Mymryk, J.S. (2014). The adenovirus 55 residue E1A protein is a transcriptional activator and binds the unliganded thyroid hormone receptor. J. Gen. Virol. *95*, 142–152.

36. Radko, S., Jung, R., Olanubi, O., and Pelka, P. (2015). Effects of adenovirus type 5 E1A isoforms on viral replication in arrested human cells. PLoS One *10*, e0140124.

37. Kleinstiver, B.P., Pattanayak, V., Prew, M.S., Tsai, S.Q., Nguyen, N.T., Zheng, Z., and Joung, J.K. (2016). High-fidelity CRISPR-Cas9 nucleases with no detectable genome-wide off-target effects. Nature *529*, 490–495.

38. Ramakrishna, S., Cho, S.W., Kim, S., Song, M., Gopalappa, R., Kim, J.S., and Kim, H. (2014). Surrogate reporter-based enrichment of cells containing RNA-guided Cas9 nuclease-induced mutations. Nat. Commun. *5*, 3378.

39. Cundy, K.C. (1999). Clinical pharmacokinetics of the antiviral nucleotide analogues cidofovir and adefovir. Clin. Pharmacokinet. *36*, 127–143.

40. Lagos-Quintana, M., Rauhut, R., Yalcin, A., Meyer, J., Lendeckel, W., and Tuschl, T. (2002). Identification of tissue-specific microRNAs from mouse. Curr. Biol. *12*, 735–739.

41. Han, J., Lee, Y., Yeom, K.H., Nam, J.W., Heo, I., Rhee, J.K., Sohn, S.Y., Cho, Y., Zhang, B.T., and Kim, V.N. (2006). Molecular basis for the recognition of primary microRNAs by the Drosha-DGCR8 complex. Cell *125*, 887–901.

42. Macias, S., Cordiner, R.A., and Cáceres, J.F. (2013). Cellular functions of the microprocessor. Biochem. Soc. Trans. *41*, 838–843.

43. Chung, K.H., Hart, C.C., Al-Bassam, S., Avery, A., Taylor, J., Patel, P.D., Vojtek, A.B., and Turner, D.L. (2006). Polycistronic RNA polymerase II expression vectors for RNA interference based on BIC/miR-155. Nucleic Acids Res. *34*, e53.

44. Xie, C., Chen, Y.L., Wang, D.F., Wang, Y.L., Zhang, T.P., Li, H., Liang, F., Zhao, Y., and Zhang, G.Y. (2017). SgRNA expression of CRIPSR-Cas9 system based on MiRNA polycistrons as a versatile tool to manipulate multiple and tissue-specific genome editing. Sci. Rep. *7*, 5795.

45. Yan, Q., Xu, K., Xing, J., Zhang, T., Wang, X., Wei, Z., Ren, C., Liu, Z., Shao, S., and Zhang, Z. (2016). Multiplex CRISPR/Cas9-based genome engineering enhanced by Drosha-mediated sgRNA-shRNA structure. Sci. Rep. *6*, 38970.

46. Kneidinger, D., Ibrišimović, M., Lion, T., and Klein, R. (2012). Inhibition of adenovirus multiplication by short interfering RNAs directly or indirectly targeting the viral DNA replication machinery. Antivir. Res. *94*, 195–207.

47. Ibrišimović, M., Kneidinger, D., Lion, T., and Klein, R. (2013). An adenoviral vector-based expression and delivery system for the inhibition of wild-type adenovirus replication by artificial microRNAs. Antivir. Res. *97*, 10–23.

48. Wang, D., Tai, P.W.L., and Gao, G. (2019). Adeno-associated virus vector as a platform for gene therapy delivery. Nat. Rev. Drug Discov. *18*, 358–378.

49. Meier, A.F., Fraefel, C., and Seyffert, M. (2020). The interplay between adeno-associated virus and its helper viruses. Viruses *12*. https://doi.org/10.3390/v12060662.

50. Fahnestock, M.L., and Lewis, J.B. (1989). Genetic dissection of the transactivating domain of the E1a 289R protein of adenovirus type 2. J. Virol. *63*, 1495–1504.

51. Jelsma, T.N., Howe, J.A., Evelegh, C.M., Cunniff, N.F., Skiadopoulos, M.H., Floroff, M.R., Denman, J.E., and Bayley, S.T. (1988). Use of deletion and point mutants spanning the coding region of the adenovirus 5 E1A gene to define a domain that is essential for transcriptional activation. Virology *163*, 494–502.

52. Douglas, J.L., and Quinlan, M.P. (1994). Efficient nuclear localization of the Ad5 E1A 12S protein is necessary for immortalization but not cotransformation of primary epithelial cells. Cell Growth Differ. *5*, 475–483.

53. Douglas, J.L., and Quinlan, M.P. (1995). Efficient nuclear localization and immortalizing ability, two functions dependent on the adenovirus type 5 (Ad5) E1A second exon, are necessary for cotransformation with Ad5 E1B but not with T24ras. J. Virol. *69*, 8061–8065.

54. Cohen, M.J., King, C.R., Dikeakos, J.D., and Mymryk, J.S. (2014). Functional analysis of the C-terminal region of human adenovirus E1A reveals a misidentified nuclear localization signal. Virology *468-470*, 238–243.

55. Köhler, M., Görlich, D., Hartmann, E., and Franke, J. (2001). Adenoviral E1A protein nuclear import is preferentially mediated by importin alpha3 in vitro. Virology *289*, 186–191.

56. Lyons, R.H., Ferguson, B.Q., and Rosenberg, M. (1987). Pentapeptide nuclear localization signal in adenovirus E1a. Mol. Cell Biol. *7*, 2451–2456.

57. Marshall, K.S., Cohen, M.J., Fonseca, G.J., Todorovic, B., King, C.R., Yousef, A.F., Zhang, Z., and Mymryk, J.S. (2014). Identification and characterization of multiple conserved nuclear localization signals within adenovirus E1A. Virology *454-455*, 206–214.

58. Standiford, D.M., and Richter, J.D. (1992). Analysis of a developmentally regulated nuclear localization signal in Xenopus. J. Cell Biol. *118*, 991–1002.

59. Schaar, K., Geisler, A., Kraus, M., Pinkert, S., Pryshliak, M., Spencer, J.F., Tollefson, A.E., Ying, B., Kurreck, J., Wold, W.S., et al. (2017). Anti-adenoviral artificial MicroRNAs expressed from AAV9 vectors inhibit human adenovirus infection in immunosuppressed Syrian hamsters. Mol. Ther. Nucleic Acids *8*, 300–316.

60. Kalesnykas, G., Kokki, E., Alasaarela, L., Lesch, H.P., Tuulos, T., Kinnunen, K., Uusitalo, H., Airenne, K., and Yla-Herttuala, S. (2017). Comparative Study of adeno-associated virus, adenovirus, baculovirus and lentivirus vectors for gene therapy of the eyes. Curr. Gene Ther. *17*, 235–247.

61. Solinís, M.Á., del Pozo-Rodríguez, A., Apaolaza, P.S., and Rodríguez-Gascón, A. (2015). Treatment of ocular disorders by gene therapy. Eur. J. Pharm. Biopharm. *95*, 331–342.

62. Gonzalez, G., Yawata, N., Aoki, K., and Kitaichi, N. (2019). Challenges in management of epidemic keratoconjunctivitis with emerging recombinant human adenoviruses. J. Clin. Virol. *112*, 1–9.

63. Rajaiya, J., Saha, A., Ismail, A.M., Zhou, X., Su, T., and Chodosh, J. (2021). Adenovirus and the cornea: more than meets the eye. Viruses *13*, 293.

64. Labun, K., Montague, T.G., Krause, M., Torres Cleuren, Y.N., Tjeldnes, H., and Valen, E. (2019). CHOPCHOP v3: expanding the CRISPR web toolbox beyond genome editing. Nucleic Acids Res. *47*, W171–W174.

65. Doench, J.G., Fusi, N., Sullender, M., Hegde, M., Vaimberg, E.W., Donovan, K.F., Smith, I., Tothova, Z., Wilen, C., Orchard, R., et al. (2016). Optimized sgRNA design to maximize activity and minimize off-target effects of CRISPR-Cas9. Nat. Biotechnol. *34*, 184–191.

66. Ma, H., Wu, Y., Dang, Y., Choi, J.G., Zhang, J., and Wu, H. (2014). Pol III promoters to express small RNAs: delineation of transcription initiation. Mol. Ther. Nucleic Acids *3*, e161.

67. Callahan, B.J., Wong, J., Heiner, C., Oh, S., Theriot, C.M., Gulati, A.S., McGill, S.K., and Dougherty, M.K. (2019). High-throughput amplicon sequencing of the full-length 16S rRNA gene with single-nucleotide resolution. Nucleic Acids Res. *47*, e103.

68. Clement, K., Rees, H., Canver, M.C., Gehrke, J.M., Farouni, R., Hsu, J.Y., Cole, M.A., Liu, D.R., Joung, J.K., Bauer, D.E., and Pinello, L. (2019). CRISPResso2 provides accurate and rapid genome editing sequence analysis. Nat. Biotechnol. *37*, 224–226.

OMTN, Volume *32*

Supplemental information

Inhibition of adenovirus replication by CRISPR-Cas9-mediated targeting of the viral E1A gene

Zrinka Didara, Florian Reithofer, Karina Zöttl, Alexander Jürets, Izabella Kiss, Angela Witte, and Reinhard Klein

Table S1: Primer List

Primer names	5´-3´
pENTR4 FW	GGAAAGAACCGGCGCGCCAAGCTTGAATTCGCGGCCGCACTC
pENTR4 RV	AAGCAGATTCGACTGAATTGGTTCCCATGGTG
CMV Tet02_FW	CAATTCAGTCGAATCTGCTTAGGGTTAG
CMV Tet02_FW	ATAGTGAGTCGTTTAAACGCTAGAGTCC
Cas9 HF1 FW	GCGTTTAAACGACTCACTATAGGGAGAGCC
Cas9 HF1 RV	TTGGCGCGCCGGTTCTTTCCGCCTCAGAAG
RV01 sequencing FW	GTTCCAGTACGGCTCCAAG
RV01 sequencing RV	CTGGCGGCCGCTTTACTTG
T7E1 E1A Set1 FW	CAGCGAGTAGAGTTTTCTCC
T7E1 E1A Set1 RW	GTAGACAAACATGCCACAGG
T7E1 E1A Set2 FW	CGGTGAGTTCCTCAAGAG
T7E1 E1A Set2 RW	CCAAACCCACCACTCTATC
T7E1 E1A Set3 FW	GTCAGCTGACGTGTAGTG
T7E1 E1A Set3 RW	CCGTACTACTATTGCATTCTCTAG
E3 qPCR FW	TGCTGCACTGCTATGCTAAT
E3 qPCR RV	TCCTCAATAAAGCTGCGTCTG
E3 qPCR probe	TGCTCGCTTTGGTCTGTACCCTAC
E1A/E1B loop seq FW	TACCCGGTGAGTTCCTCAAG
E1A/E1B loop seq RV	GCACCCATCCCAGCTTAACC
Cas9 qPCR FW	ACGGGATAAGAGACAAGCAAAG
Cas9 qPCR RV	GGTTAAAGAGTCATCATGGATCAG
Cas9HFqPCR_probe	TAAAGAGCGACGGCTTCGCCAATA
OTUD5 F1	ATGAGAGAGAGTGCAGGGGT
OTUD5 R1	TCACAGGCCTAGCAGATCCT
IFFO1 F1	GCTCCCACCTCCCTTCAAAA
IFFO1 R1	GTGGGTGTCTGCTACTTCCC
GPR85 F1	GTGTGCTCAGTCCAAGAGGG
GPR85 R1	TGAGGAGTCAAGAGCAACGG
UNC80 F2	GCCATTTTCAGAGGAGGACA
UNC80 R2	ACGTATGCAAGAGGACACACC
RNF111 F1	CGTGGCACTATCACATTATTTACAG
RNF111 R1	GTCTTTCCACAGGGCAAGCA
FOXJ2 F2	GGAGAGGCCACATTACCAAG
FOXJ2 R2	TTGTCCTCATGCCCTACCAT
CTD-3060P21.1/RAP1GAP2 F1	CTGCCTGCTGCTGTCTTAGT
CTD-3060P21.1/RAP1GAP2 R1	TCCGGAATCTGCCCTCAGTA

TOB1-AS1 F1	GTCTGGAAAGGGACTGTGGG
TOB1-AS1 R1	TAAACGGATCCGAGTCGCAG
ABCF3 F1	CCTGAGGAGGAGTACCGTCA
ABCF3 R1	CTGAAAGACAGGAGGGCAGG
TMEM110 F1	CACCACCACCTGGATCTCAC
TMEM110 R1	TGAGAGCAGATCAGAGGGCT
PRKACB F1	CCACCATGGTGTTCTGGAGG
PRKACB R1	TGTAGCATCAAAAGAAGGCCA
RP5-964N17.1 F1	TGCTGTCTAAGTTCTGCGCC
RP5-964N17.1 R1	GCCCAGTCAGCTACAGAGTG

Table S2: Surrogate Reporter Vector Target Sequences

Vector name	Target sequence inserted into RV01	5´-3´
RVO1-1,3,4	E1A gRNA 1,3,4	GAATTCAACCTTGTACCGGAGGTGATCGATCTTACCTG CCACGAGGCTGGCTGGATCC
RVO1-2,9	E1A gRNA 2,9	GAATTCCCCCCGAAGATCCCAACGAGGAGGCGTGTAA TGTTGGCGGTGCAGGAAGGGAGGATCC
RVO1-5,6,8	E1A gRNA 5,6,8	GAATTCGCCAGTCTTTTGGACCAGCTGATCGTCCACCT CCTAGCCATTTTGAACCACCTACGGATCC
RVO1-7,10	E1A gRNA 7,10	GAATTCGCCCGGTTCTCCGGAGCCGCCTCACCTTTCC CGGCAGCCCGAGCAGCCGGGATCC

Figure S1

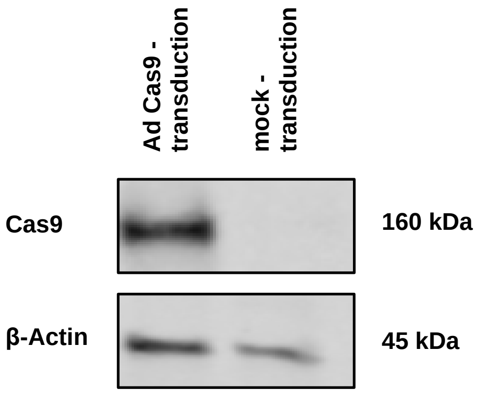

Figure S1. Expression of spCas9-HF1.

The functionality of the spHF-Cas9 expression cassettes harboring the doxycycline-regulatable CMV promoter which is present in all adenoviral CRISPR/Cas9 vectors was proven in the absence of doxycycline in HeLa cells lacking the tetracycline repressor. Equal amounts of protein from HeLa cells at 48 h post-transduction were subjected to Western blot analysis for the detection of spHF-Cas9 and for comparison of □-actin. The expression is exemplarily shown for the vector expressing only spCas9-HF1.

Figure S2

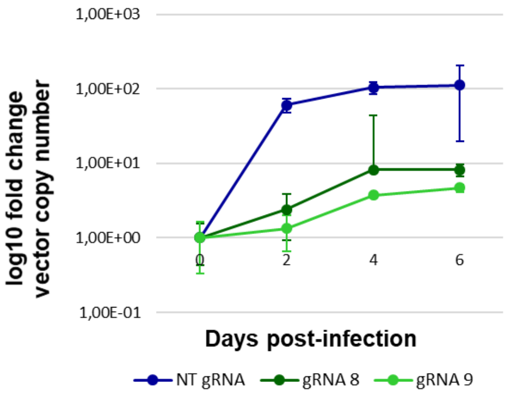

Figure S2. Replication of the adenoviral vectors in HAdV-5-infected cells.

HeLa cells were transduced with the adenoviral vectors containing Cas9 in combination with the targeting gRNAs 8 or 9 or with a non-targeting (NT) gRNA at an MOI of 30. 24 h after transduction the cells were infected with HAdV-5 at an MOI of 0.1. Vector copy numbers at time points 0, 2, 4, and 6 days post-infection were determined by qPCR with primers/probe specific for the Cas9-encoding part of the vectors. Data represent the means (n = 3) ± SD of triplicate infections of a representative experiment.

Figure S3

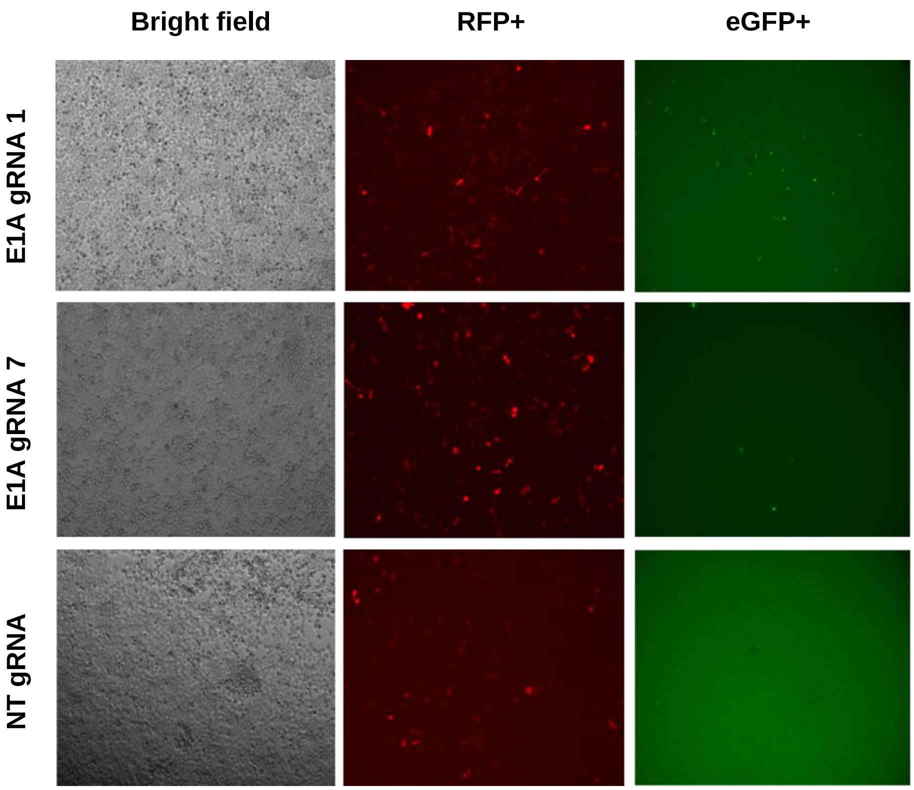

Figure S3. Detection of gene editing by E1A-targeting gRNAs 1 and 7 in surrogate reporter assays.

HEK293 cells were transfected with the surrogate reporter vector and transduced with the recombinant adenoviral vectors expressing either a targeting or a non-targeting (NT) gRNA. Fluorescence was monitored 48h post-transduction with a Leica DMi8 System. Bright field, red and green fluorescence images at a magnification of 10x are shown. Microscopy settings were: HC PL FLUOTAR CS 10x/0.40 DRY; Camera Leica DFC 360FX: active resolution 1392 x 1040, pixel bitdepth 12/8 bit, pixel size 6.45 µm x 6.45 µm; live image with 1392 x 1040 at 20 images/second.

Figure S4

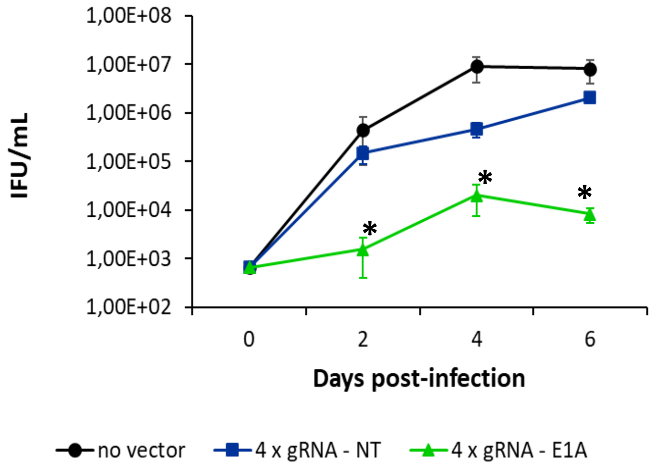

Figure S4. Inhibition of HAdV-5 replication in A549 cells.

A549 cells were transduced with the adenoviral vector containing Cas9 in combination with gRNAs 1, 7, 8, and 9 expressed from individual promoters or with a control vector carrying four non-targeting (NT) gRNAs at an MOI of 100 followed by infection of the cells with HAdV-5 at an MOI of 0.01 24 h later. Numbers of infectious virus particles were determined at the indicated time points and were expressed as IFU/mL (infectious units per mL). Data represent the means (n = 3) ± SD of three infections. *$p < 0.05$; *** $p < 0.001$.

Figure S5

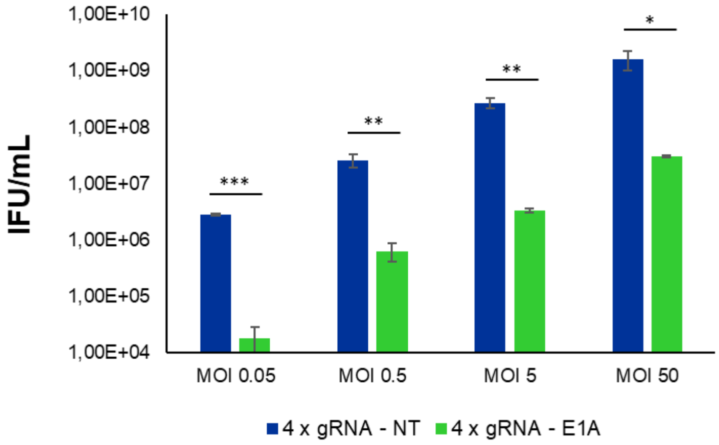

Figure S5. Inhibition of HAdV-5 replication at increased MOIs.

A549 cells were transduced with the adenoviral vectors containing Cas9 in combination with the E1A-targeting gRNAs 1, 7, 8, and 9 expressed from individual promoters or with a corresponding control vector carrying four non-targeting (NT) gRNAs instead of the targeting gRNAs at a constant MOI of 50. 24 h after transduction the cells were infected with HAdV-5 at increasing MOIs ranging from 0.05 to 50. 48 h after infection numbers of infectious virus particles were determined and were expressed as IFU/mL (infectious units per mL). Data represent the means (n = 3) ± SD of triplicate infections of a representative experiment. *$p < 0.05$; ** $p < 0.01$; *** $p < 0.001$.

Figure S6

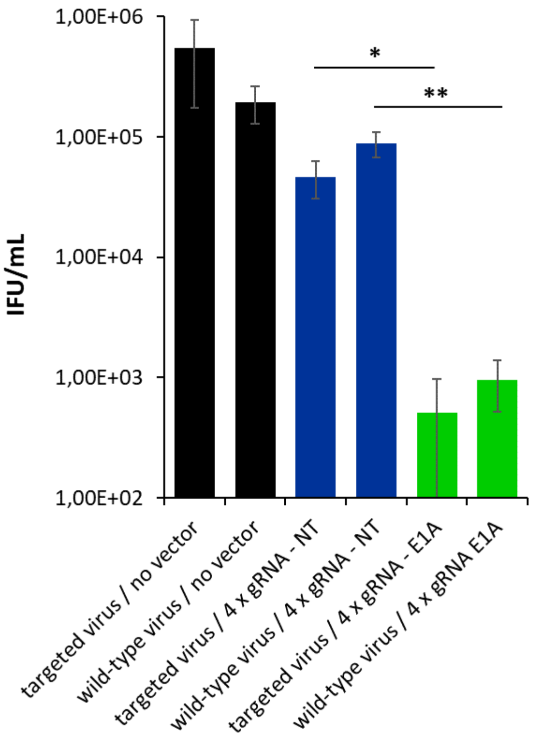

Figure S6. CRISPR/Cas9-mediated inhibition of replication of virus recovered from the first round of targeting by CRISPR/Cas9

HeLa cells were transduced with the adenoviral vector containing Cas9 in combination with gRNAs 1, 7, 8, and 9 expressed from individual promoters (green), with a control vector carrying four non-targeting (NT) gRNAs (blue), or were mock-transduced (black) at an MOI of 100. 24 h later cells were infected with virus recovered after the first round of targeting by CRISPR/Cas9 (day 6 time point; virus pooled from three independent experiments) or with non-targeted wild-type virus at an MOI of 0.01. Virus was allowed to replicate for two days. At day 2 post-infection numbers of infectious virus particles were determined and were expressed as IFU/mL (infectious units per mL). Data represent the means (n = 3) ± SD of three infections. *$p < 0.05$, **$p < 0.01$.

Figure S7

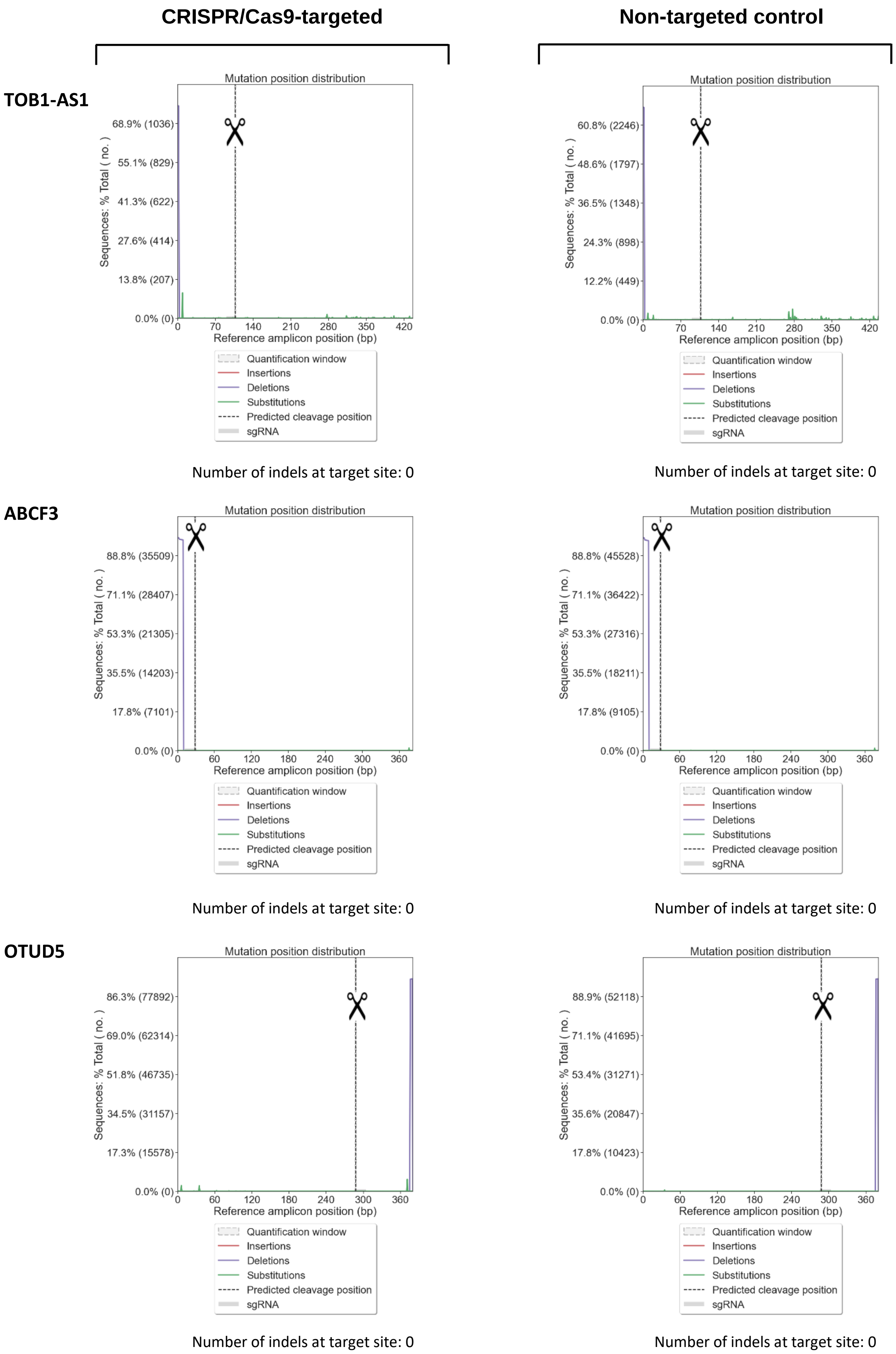
CRISPR/Cas9-targeted
Non-targeted control
TOB1-AS1
ABCF3
OTUD5
Mutation position distribution
Sequences: % Total (no.)
Reference amplicon position (bp)
68.9% (1036)
55.1% (829)
41.3% (622)
27.6% (414)
13.8% (207)
0.0% (0)
60.8% (2246)
48.6% (1797)
36.5% (1348)
24.3% (898)
12.2% (449)
88.8% (35509)
71.1% (28407)
53.3% (21305)
35.5% (14203)
17.8% (7101)
88.8% (45528)
71.1% (36422)
53.3% (27316)
35.5% (18211)
17.8% (9105)
86.3% (77892)
69.0% (62314)
51.8% (46735)
34.5% (31157)
17.3% (15578)
88.9% (52118)
71.1% (41695)
53.4% (31271)
35.6% (20847)
17.8% (10423)
Quantification window
Insertions
Deletions
Substitutions
Predicted cleavage position
sgRNA
Number of indels at target site: 0

Figure S7 - continued

CRISPR/Cas9-targeted | **Non-targeted control**

IFFO1

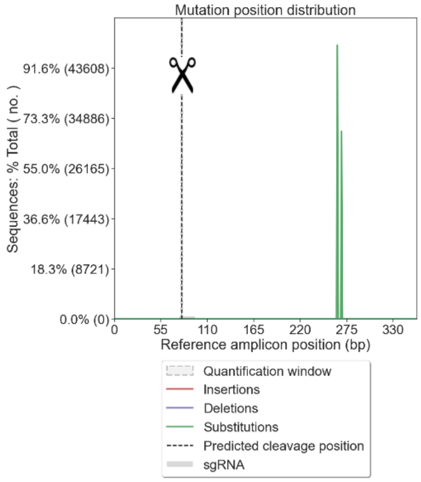

Number of indels at target site: 0

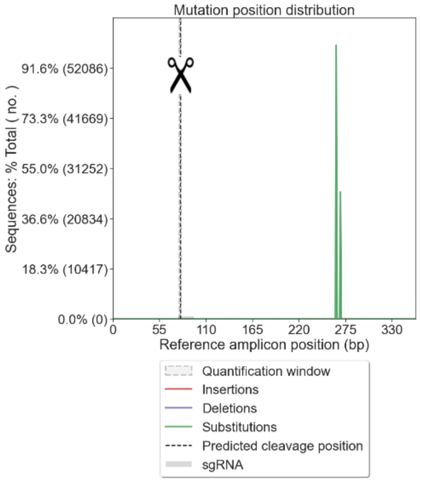

Number of indels at target site: 0

RP5-964N17.1

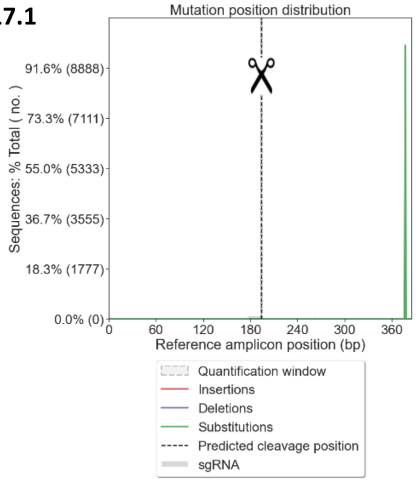

Number of indels at target site: 0

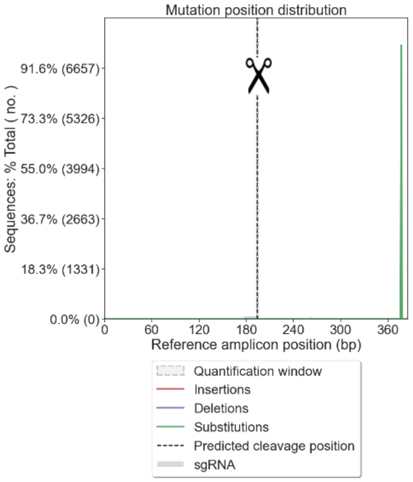

Number of indels at target site: 0

GPR85

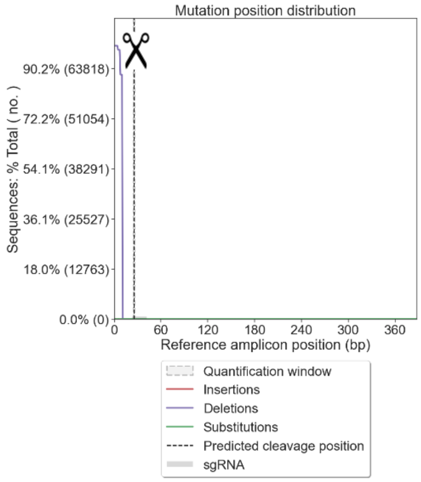

Number of indels at target site: 0

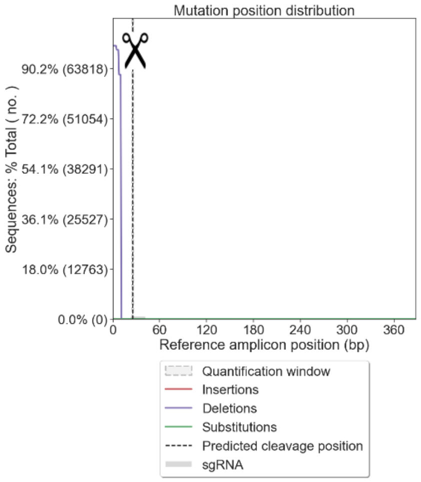

Number of indels at target site: 0

Figure S7 - continued

CRISPR/Cas9-targeted | **Non-targeted control**

PRKACB

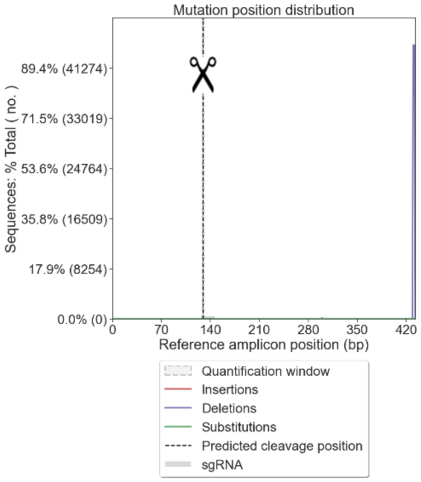

Number of indels at target site: 0

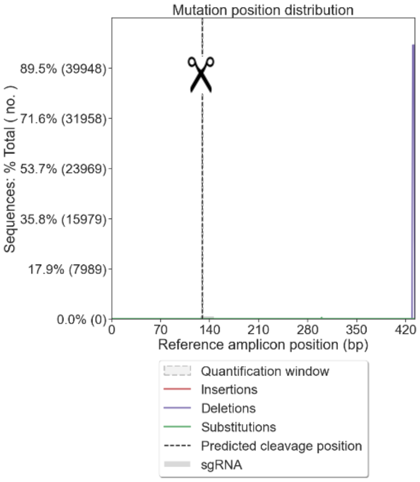

Number of indels at target site: 0

TMEM110

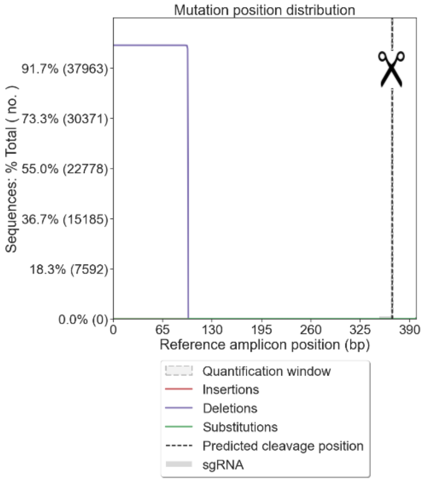

Number of indels at target site: 0

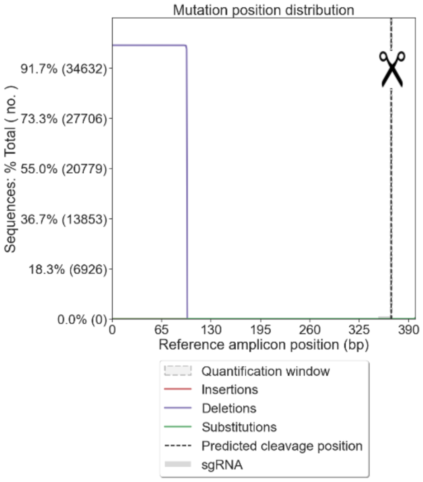

Number of indels at target site: 0

UNC80

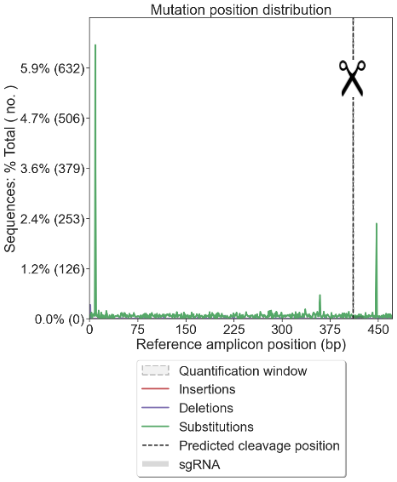

Number of indels at target site: 0

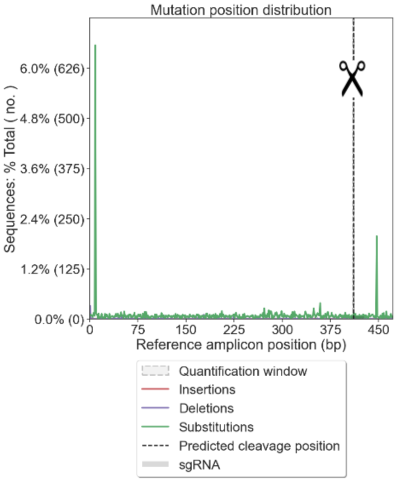

Number of indels at target site: 0

Figure S7 - continued

	**CRISPR/Cas9-targeted**	**Non-targeted control**
**RNF111**	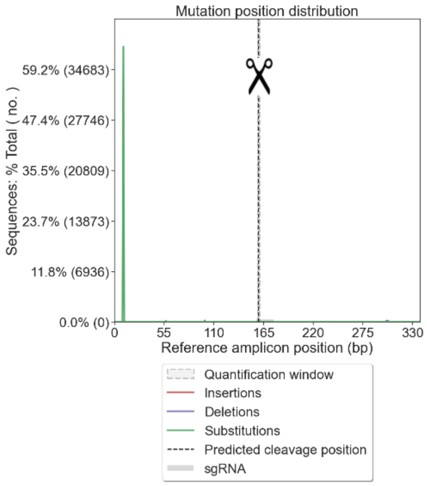 Number of indels at target site: 0	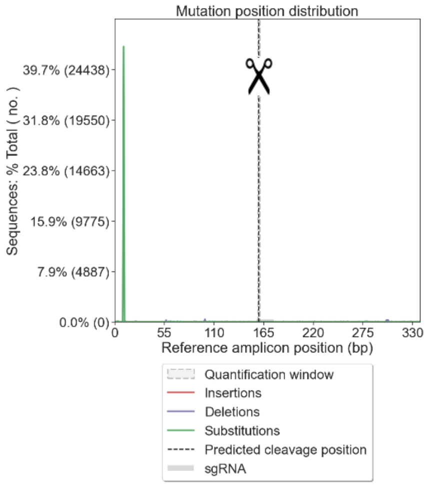 Number of indels at target site: 0
**FOXJ2**	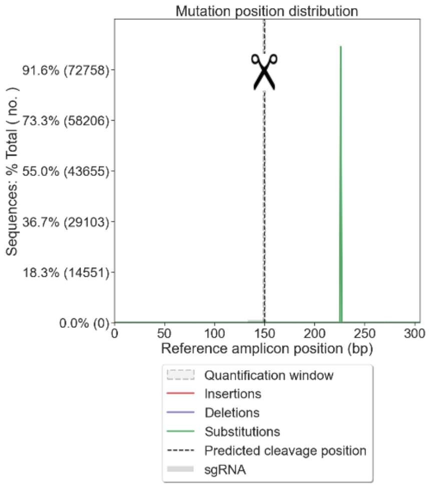 Number of indels at target site: 0	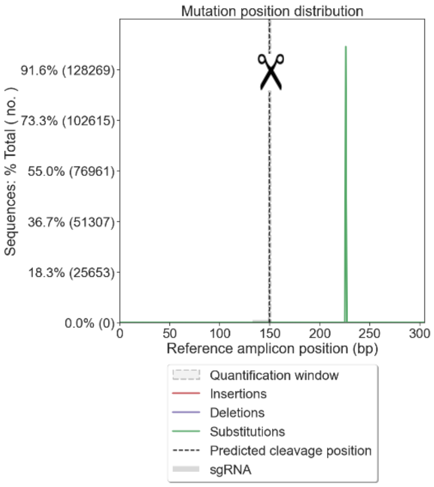 Number of indels at target site: 0
**CTD-3060P21.1/ RAP1GAP2**	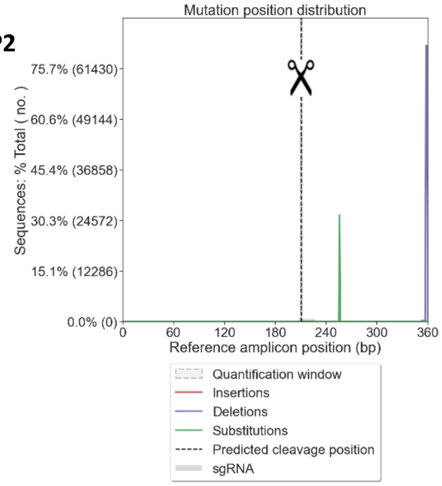 Number of indels at target site: 0	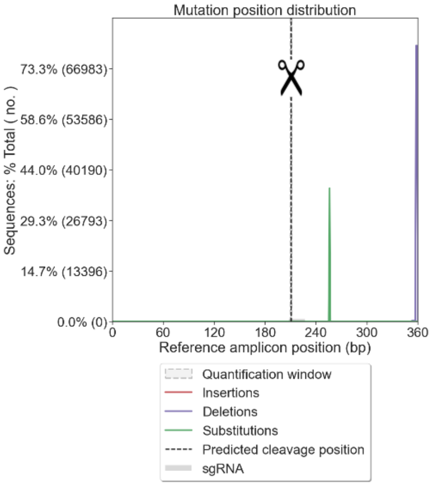 Number of indels at target site: 0

Figure S7. CRISPR/Cas9-mediated cleavage at potential off-target sites.

HeLa cells were transduced with the adenoviral vectors harboring Cas9 in combination with the four gRNAs 1, 7, 8 and 9 expressed from separate promoters at an MOI of 100 or were mock-transduced. 24 h after transduction the cells were infected with HAdV-5 at an MOI of 0.01. Two days post-infection DNA was isolated from the cells, the DNA regions comprising the potential off-target sites were amplified by PCR, the amplicons were subjected to next generation sequencing, and the sequencing reads were mapped to the respective reference amplicon sequences. Percentages of modifications around the indicated potential off-target sites in cells targeted with CRISPR/Cas9 (left) and in non-targeted control cells (right) representing background noise as a result of randomly occurring PCR amplification/sequencing were calculated with CRISPResso2. Insertions, deletions, and substitutions are indicated. Deletions (blue) appearing close to the borders of the graphs do not represent CRISPR/Cas9-mediated modifications but indicate the ends of the amplicons that were not covered by sequencing. Substitutions (green) occurring at more or less the same frequencies at the same positions in targeted and non-targeted cells represent mutations that have accumulated in the HeLa cell population that was used for the experiments. The potential cleavage sites at which modifications would appear in case of off-targeting are indicated with dashed lines and pairs of scissors.
